# Cellular depletion of major cathepsin proteases reveals their concerted activities for lysosomal proteolysis

**DOI:** 10.1007/s00018-024-05274-4

**Published:** 2024-05-22

**Authors:** Lisa Gallwitz, Florian Bleibaum, Matthias Voss, Michaela Schweizer, Katharina Spengler, Dominic Winter, Frederic Zöphel, Stephan Müller, Stefan Lichtenthaler, Markus Damme, Paul Saftig

**Affiliations:** 1 Institute of Biochemistry, Christian-Albrechts-University Kiel, Olshausenstr. 40, 24098 Kiel, Germany; 2 Center for Molecular Neurobiology (ZMNH), UKE, Falkenried 94, 20251 Hamburg, Germany; 3 Institute for Biochemistry and Molecular Biology, Medical Faculty, University of Bonn, Bonn, Germany; 4 German Center for Neurodegenerative Diseases (DZNE), München, Feodor-Lynen-Str. 17, 81377 Munich, Germany; 5 Neuroproteomics, School of Medicine, Klinikum rechts der Isar, Technical University of Munich, 81675 Munich, Germany; 6 Munich Cluster for Systems Neurology (SyNergy), Munich, Germany

**Keywords:** Cathepsins, Lysosome, Autophagy, Bulk proteolysis, Proteolysis, Amyloid precursor protein, Proteomics, Protease network

## Abstract

Proteins delivered by endocytosis or autophagy to lysosomes are degraded by exo- and endoproteases. In humans 15 lysosomal cathepsins (CTS) act as important physiological regulators. The cysteine proteases CTSB and CTSL and the aspartic protease CTSD are the most abundant and functional important lysosomal proteinases. Whereas their general functions in proteolysis in the lysosome, their individual substrate, cleavage specificity, and their possible sequential action on substrate proteins have been previously studied, their functional redundancy is still poorly understood. To address a possible common role of highly expressed and functional important CTS proteases, we generated CTSB-, CTSD-, CTSL-, and CTSBDL-triple deficient (KO) human neuroblastoma-derived SH-SY5Y cells and CTSB-, CTSD-, CTSL-, CTSZ and CTSBDLZ-quadruple deficient (KO) HeLa cells. These cells with a combined cathepsin deficiency exhibited enlarged lysosomes and accumulated lipofuscin-like storage material. The lack of the three (SH-SY5Y) or four (HeLa) major CTSs caused an impaired autophagic flux and reduced degradation of endocytosed albumin. Proteome analyses of parental and CTS-depleted cells revealed an enrichment of cleaved peptides, lysosome/autophagy-associated proteins, and potentially endocytosed membrane proteins like the amyloid precursor protein (APP), which can be subject to endocytic degradation. Amino- and carboxyterminal APP fragments accumulated in the multiple CTS-deficient cells, suggesting that multiple CTS-mediated cleavage events regularly process APP. In summary, our analyses support the idea that different lysosomal cathepsins act in concert, have at least partially and functionally redundant substrates, regulate protein degradation in autophagy, and control cellular proteostasis, as exemplified by their involvement in the degradation of APP fragments.

**Supplementary Information:**

The online version contains supplementary material available at 10.1007/s00018-024-05274-4.

## Introduction

Cellular proteins are subject to proteolysis as they eventually get co- and post-translationally hydrolyzed or degraded. Proteolytic intracellular hotspots are the proteasome and the lysosome, with its full set of proteolytic enzymes. In eukaryotes, these two major pathways are independent but inter-connected processes [1]. Lysosomes mainly degrade long-lived proteins, insoluble aggregates, macromolecular compounds, entire organelles, and pathogens after delivery by endocytosis, phagocytosis, or macroautophagy [1, 2]. Particularly under conditions of starvation, lysosomal proteolysis is critical for amino acid recycling for newly synthesized proteins by degrading parts of the cytosol and damaged or aged organelles like mitochondria via the autophagy pathway [3].

The most abundant hydrolases residing in the lysosome belong to the family of cathepsins (CTSs) [4, 5]. CTSs are differentially expressed among cell types and tissues and can be divided into aspartic-, cysteine- and serine-type CTSs based on their catalytic mechanism [6, 7]. In humans, the different lysosomal CTSs act as essential regulators in physiological processes like digestion, autophagy, innate immunity, proliferation, or apoptosis [5, 7]. Synthesized in the endoplasmic reticulum (ER) as inactive pre-pro-enzymes, CTSs are subjected to several proteolytic cleavage events to become fully active and matured en route to the lysosome [6].

The cysteine proteases CTSB and CTSL and the aspartic protease CTSD are important lysosomal proteinases and play critical functions in the last steps of autophagy as well as lysosomal and neurodegenerative diseases [8–11]. Both CTSB and CTSL are endopeptidases, while CTSB also presents peptidyl-dipeptidase activity [12, 13]. CTSB and CTSL have redundant functions, reflected by a severe neurological phenotype and premature death of *Ctsb/Ctsl* double knockout mice in the first month of life, while single knockouts have a normal life span and show no major neurological symptoms [14, 15]. CTSD is an aspartic protease that constitutes (depending on the cell type) about 10% of the total soluble lysosomal proteins, with concentrations measured in liver lysosomes reaching up to 0.7 mM [16]. Though expressed in almost all cell types and tissues, the enzyme is mainly found within the brain, lung, and gall bladder [17]. CTSD is a lysosomal endopeptidase stable at an acidic pH [18, 19] and preferably cleaves substrates between Phe-Phe bonds [20, 21]. In addition, other amino acids like tyrosine, leucine, and methionine are favored at the P1 position [20], i.e., the first amino acid residue after the scissile bond. In mice, CTSD deficiency leads to a severe phenotype characterized by seizures, neurodegeneration, and early death after approximately three to four weeks [22]. Additionally, the lack of CTSD causes an impairment in autophagic flux, resulting in the accumulation of neuronal storage material, including lipofuscin and mature saposins [9]. Interestingly, exogenously added CTSL could at least in part clear that accumulated proteinaceous material such as saposin or repair the autophagic impairments in *Ctsd* knockout neuronal stem cell-derived astrocytes and mice, a model for neuronal ceroid lipofuscinosis [23], suggesting a compensatory function between CTSD and CTSL. CTSZ is another abundant lysosomal cysteine protease with unique carboxypeptidase activity and its relation with other CTSs has been investigated [24]. Redundant functions of CTSB and CTSZ have been described in tissue homeostasis and in cancer [25].

It is not well understood if and how lysosomal CTSs act in concert and function in a hierarchical and/or in a redundant manner. Despite the contribution of CTSs in lysosomal proteolysis, there is a need to understand how individual CTSs are functionally embedded in the network of lysosomal proteases and protein degradation pathways. It is also of note that the selected CTS proteases are also involved in extra-lysosomal processing pathways such as programmed cell death [26] and extracellular matrix remodelling [27].

In this study, detailed investigations of multiple CTS-depleted cell lines revealed enlarged lysosomes and accumulation of autofluorescent lysosomal storage material. Such a phenotype was only evident in the combined but not in the single knockout cell lines. The combined deficiency of the major CTSs also revealed a reduction in autophagic flux and a slower degradation of endocytosed serum albumin. A difference in protein and semi-tryptic peptide abundance was observed by proteome analysis of whole-cell lysates from wildtype and CTS-depleted cells. Moreover, a focussed quantitative and qualitative analysis of semi-tryptic peptides in our proteome data stemming from ongoing cellular cleavage processes revealed altered proteolytic processing of proteins in CTS-depleted cells. The homeostasis of the amyloid precursor protein (APP) and its amino- and carboxy-terminal fragments, which are known to aggregate under conditions of lysosomal dysfunction, including defective lysosomal proteolysis [28, 29] was regulated by CTS-mediated proteolytic processing. With this study, we provide evidence for a concerted activity of major cathepsins in two different cell types. It also provides a cellular tool to address questions about how cathepsins are involved in the degradation of selected proteins and how cathepsins may regulate cellular metabolism.

## Results

### Expression of major lysosomal proteinases in HeLa and SH-SY5Y cells

**Fig. 1:**
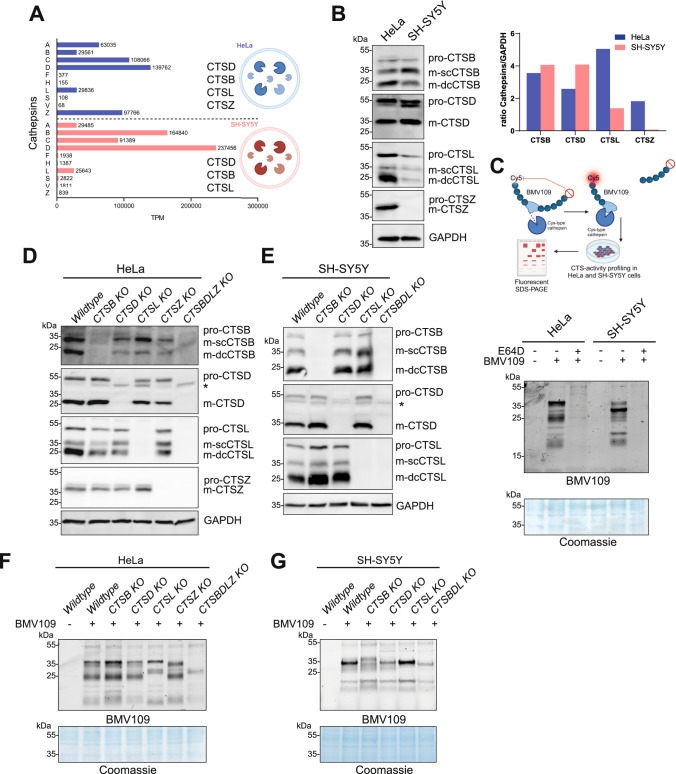
Analysis of the endogenous expression and activity of cathepsins in HeLa and SH-SY5Y cells. **A** Previously published [75] RNA sequencing data from HeLa and SH-SY5Y cells, publicly available through the Human Proteome Atlas portal, show transcripts per million (TPM) for 10 human cathepsins. **B** Immunoblot and protein expression analysis of endogenous CTSB, CTSD, CTSL and CTSZ expression in HeLa and SH-SY5Y wildtype (WT) cells. The signal intensities of all detected bands with respective CTS-antibodies were normalized to GAPDH. **C** The quenched cysteine-protease activity-based probe (ABP) BMV-109 contains a fluorophore-quencher pair. Once the ABP binds covalently to the active-site cysteine, the quenching group is released, and the protease is irreversibly labelled, resulting in the emission of fluorescence that can be detected via fluorescent SDS-PAGE. The in-gel fluorescence emission of the scanned SDS-PAGE shows BMV-109-labelled HeLa and SH-SY5Y WT cells treated with or without 50 μM E64D (cysteine-protease inhibitor). Cy5 signal was detected at excitation at 635 nm. Total protein staining with Coomassie Brilliant Blue was used as a loading control. **D** The immunoblot shows a successful generation of two HeLa CTSB, CTSD, CTSL, CTSZ, and CTSBDLZ KO clones (**D**) and of SH-SY5Y CTSB, CTSD, CTSL and CTSBDL clones (**E**) each. **F** Fluorescent SDS-PAGE of BMV-109-labelled HeLa (**F**) and SH-SY5Y (**G**) WT, single KO, and multiple CTS-deficient cells. *kDa* kilodalton, * indicates non-specific-binding of the antibody, *p* pro, *m* mature. sc-single chain, dc-double chain (**A**, **C**) “Created with BioRender.com.”

Since lysosomal proteinases are differentially expressed in tissues and cell types, we first surveyed transcriptome data for cathepsin mRNA levels from five established cell lines. From this list, we decided on an in-depth analysis of human cervical cancer (HeLa) and neuroblastoma (SH-SY5Y) as commonly used standard cell lines. A comparison of CTS expression in HeLa and SH-SY5Y cells revealed that 14 out of 15 cathepsins are expressed, although with a different abundance, in both cell lines [24]. The highest expressed cathepsins in HeLa cells are *CTSA*, *CTSC*, *CTSD*, *CTSL,* and *CTSZ*. In SH-SY5Y cells, the CTSs with the highest expression are *CTSA*, *CTSB*, *CTSC*, *CTSD*, and *CTSL*. The endopeptidases CTSB, CTSD, and CTSL (SH-SY5Y), and in addition, CTSZ (HeLa) cleave their substrates within their peptide backbone, resulting in smaller peptides [12, 30, 31], initiating lysosomal proteolysis [32, 33]. Given their high expression, CTSB, CTSD, CTSL, and CTSZ were selected for further knockout experiments in SH-SY5Y and HeLa cells. CTSB, CTSL and CTSD were chosen due to their high expression, known function in bulk proteolysis and their reported functional importance such as cell growth and in diseases like cancer and neurodegeneration [34, 35] and neurodegeneration [36–38]. CTSA was not chosen due to its additional non-proteolytic function as a chaperoning protein for the lysosomal glycosidases GLB1 and NEU1 [39]. Immunoblot analysis was performed to confirm transcriptome data and directly compare the relative expression between SH-SY5Y and HeLa cells (Fig. 1B).

To also analyze CTS activity in both cell lines, the pan-reactive cysteine quenched activity-based probe (qABP) BMV-109 was used [40]. Once BMV-109 is covalently bound to a cysteine-type protease, the quencher is removed, and Cy5 fluorescence can be measured following excitation at 635 nm. Labeling in living cells followed by cell lysis, SDS-PAGE, and in-gel fluorescence detection revealed different signals and signal intensities for active cysteine-type proteases in HeLa and SH-SY5Y cells supporting the differential expressions of CTSs. The cell-permeable cysteine-protease inhibitor E64D led to a complete loss of Cy5-signal in lysates of both HeLa and SH-SY5Y cells (Fig. 1C), confirming the cysteine-type protease specificity of the ABP. Of note, CTSD activity in both cell types was also demonstrated using a quenched fluorogenic CTSD substrate (Suppl. Fig. 1A).

### CRISPR/Cas9-mediated generation of single and multiple cathepsin- deficient HeLa and SH-SY5Y cells

Using CRISPR/Cas9-mediated gene editing, we obtained -after single cell selection- independent clones of viable single and multiple CTS-depleted cells, as confirmed by immunoblot analyses of the respective HeLa (Fig. 1D) and SH-SY5Y cell lysates (Fig. 1E). A loss of all forms of CTSB, CTSD, and CTSL in the triple SH-SY5Y cells and all forms in the HeLa cells (including CTSZ) was evident. To confirm the loss of their protease activity, the cysteine-protease ABP BMV-109 was applied to wildtype, single CTS KO, and multiple CTS KO cells. According to the work of Verdoes et al. and in agreement with the mRNA abundance (Fig. 1A), the major fluorescence signals represent mature forms of CTSB, CTSL, and CTSZ [40]. In the SH-SY5Y and HeLa multiple KO (Fig. 1F, G), an apparent reduction of the prominent signals was observed, indicating a significant loss of the overall cysteine proteinase activities in these cells. Cathepsin D activity was also lost in these cells (Suppl. Fig. 1B, C). Interestingly, the single KO cells displayed only modest changes in the cysteine-type specific fluorescent banding pattern, and a significant loss of proteolytic activity was observed only in the multiple CTS-KO.

### Multiple cathepsin deficiency causes increased LAMP1 levels and enlarged lysosomes

**Fig. 2:**
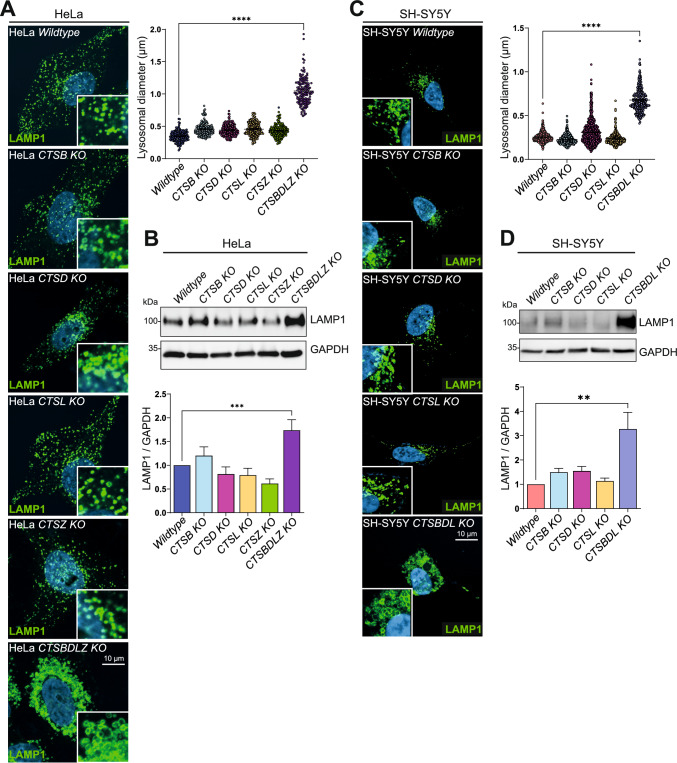
Multiple CTS-deficiency leads to enlarged lysosomes. **A** Immunostainings of lysosomal LAMP1 in HeLa WT and one of each CTSB, CTSD, CTSL, CTSZ single KO, and CTSBDLZ-multiple KO cells. Scale bar: 10 μm. The analysis of the lysosomal diameter (µm) in the multiple CTS-deficient mutant reveals a significantly increased diameter as compared to WT lysosomes. The graph depicts the lysosomal diameter in μm derived from immunostainings of LAMP-1-positive vesicles from three individually stained experiment data sets. Data represent the mean ± SEM. ****p < 0.0001. **B** The HeLa CTSBDLZ-deficient cells significantly express more LAMP1 compared to WT or single CTS-deficient cells. **C** Immunostainings of endogenous LAMP1 in SH-SY5Y WT, CTSB-, CTSD-, CTSL- and CTSBDL-deficient cells. Scale bar: 10 μm Analysis of the lysosomal diameter in SH-SY5Y WT, CTSB KO, CTSD KO, CTSL KO, CTSBDL KO cells. The graph depicts the lysosomal diameter in μm derived from immunostainings of LAMP1-positive vesicles from three individually stained experiment data sets. **D** The SH-SY5Y CTSBDL-deficient cells significantly express more LAMP1 compared to WT or single CTS-deficient cells. Data represent the mean ± SEM, one-way ANOVA with Bonferroni's multiple comparison test. ****p < 0.0001

The generation of several individual single and multiple CTS-deficient cell lines presents an exciting tool to study the resulting effects in lysosomes, i.e., the analysis of the lysosomal functions of degradation, involvement of CTSs in autophagy, and recycling of amino acids, proteins, and peptides. To analyze if lysosome morphology is changed when the major CTSs are missing, HeLa wildtype, CTSB-, CTSD-, CTSL-, CTSZ, and CTSBDLZ-deficient cells, as well as SH-SY5Y wildtype, CTSB-, CTSD, CTSL- and CTSBDL-deficient cell lines were analyzed by immunofluorescence staining using an antibody directed against LAMP1, an abundant lysosomal membrane protein (Fig. 2A–D). Comparison of the lysosomal morphology of the CTS-deficient cell lines with WT control cells revealed that the single KOs did not show obvious phenotypic changes in regard to size, distribution, or number of lysosomes, suggesting compensation by other CTSs. Importantly, CTSBDL-triple KO SH-SY5Y cells and CTSBLDZ-quadruple KO HeLa cells were characterized by a significant increase in lysosomal size (Fig. 2A, C). In addition to the phenotypic changes in lysosomal morphology of the multiple CTS-deficient cell lines, immunoblot analysis revealed a significant ~ 3.3-fold increase of LAMP1 protein expression in the SH-SY5Y CTSBDL KO cells and a 1.7-fold increase in the HeLa CTSBDLZ KO cells compared to the corresponding WT cells (Fig. 2B, D). Interestingly, the single KO cell lines did not express significantly more LAMP1 than WT cells.

### Multiple CTS-deficient lysosomes present increased autofluorescence, accumulate electron-dense storage material, and show a reduced capacity to degrade endocytosed proteins

**Fig. 3:**
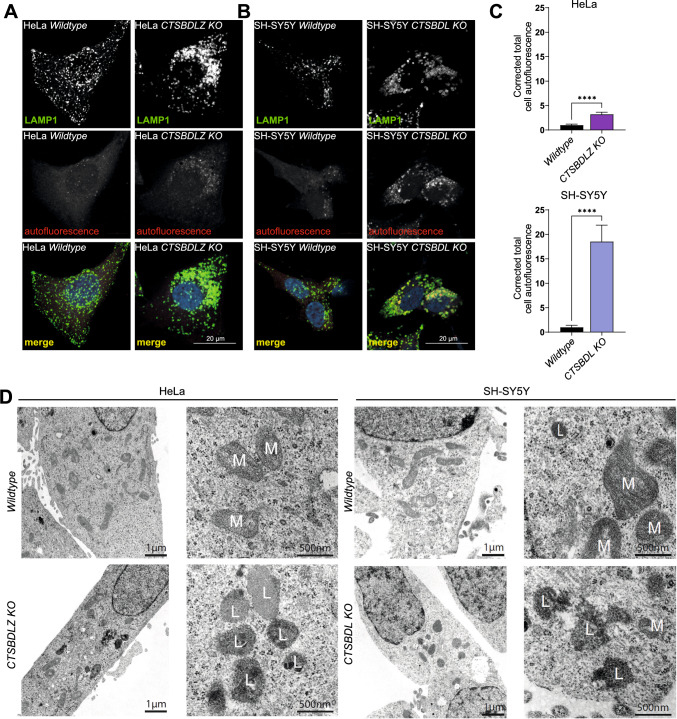
Enlarged lysosomes of multiple CTS-deficient HeLa and SH-SY5Y cells are filled with autofluorescent and electron-dense storage material. Immunostainings of **A** HeLa and **B** SH-SY5Y WT and multiple CTS-deficient cells stained with LAMP1 (green at 488 nm). Stainings were analyzed at the excitation wavelength of 594 nm to detect autofluorescent signals of lipofuscin (autofluorescence, red). Scale bar: 10 μm. **C** Corrected total cell autofluorescence signal in the HeLa and SH-SY5Y WT and multiple CTS-deficient cells. Data represent the mean ± SEM. ***p < 0.0001. **D** Transmission electron microscopy images of HeLa and SH-SY5Y WT and multiple CTS-deficient cells. Note the enlarged lysosomes filled with electron-dense storage material in the KO cell lines. *L* Lysosome, *M* Mitochondrium

Dysfunctional lysosomal proteolysis often leads to the accumulation of lipofuscin, a highly oxidized cross-linked aggregated protein, carbohydrate and lipid mixture with fluorescence emission at 570 nm to 605 nm, which also accumulates during aging [41]. Therefore, LAMP1 immunofluorescence staining of HeLa and SH-SY5Y CTS-deficient cell lines was conducted, and the samples were investigated for autofluorescence at an emission wavelength of 594 nm (Fig. 3A, B). Fluorescence microscopy of HeLa and SH-SY5Y wildtype (Fig. 3A, B) and the single CTS-KO cells (Suppl. Fig. 1D) did not show autofluorescence signals at 594 nm. In the HeLa CTSBLDZ-quadruple KO and SH-SY5Y CTSBDL-triple KO cell lines, almost all lysosomes were enlarged and filled with lipofuscin with prominent autofluorescence at 594 nm (Fig. 3A–C). Interestingly, in the neuroblastoma cell line, this effect was more pronounced, similar to prominent lipofuscin storage observed in the CNS of murine models of cathepsin deficiency [38, 42].

Enlarged lysosomes are a key feature in lysosomal storage disorders (LSDs), where undigested material accumulates within these organelles [9]. The next aim was to address whether the enlarged lysosomes of the multiple CTS-deficient cells were similarly filled with undigested cargo and which ultrastructure these lysosomes exhibit. SH-SY5Y/HeLa WT and CTSBDL-/CTSBDLZ-deficient cells were analyzed by transmission electron microscopy (TEM) (Fig. 3D). In TEM images of WT cells, lysosomes appeared as defined, round organelles filled with electron-dense structures. Multiple-CTS-deficient cells had expanded electron-dense vesicles indicated as lysosomes (L) with a more structured contour when compared to lysosomes in the SH-SY5Y WT cells.

To evaluate if multiple CTS-depleted cells can degrade endocytosed proteins properly, we applied an exogenous protease substrate, DQ-red-BSA, to wildtype and CTS-depleted cells. Upon endocytosis and lysosomal proteolysis, the quenching is relieved, and a bright fluorescent signal is emitted. As a control, bafilomycin A1 (Baf A1) treatment reduced this staining since lysosomal proteases are inactivated due to blocked acidification of the compartment. Importantly, in wildtype cells, we observed an intense vesicular fluorescence staining pattern, which was significantly reduced in HeLa and even more prominently decreased in SH-SY5Y cathepsin-multiple-deficient cells (Suppl. Fig. 2A, B). This was also confirmed biochemically in an in-gel fluorescence assay after incubation of cells with fluorescently labelled BSA-647 followed by chase times up to 16 h. In triple CTS-depleted SH-SY5Y cells, full-length BSA hardly disappeared over the time of 16 h. An additional observation was a prominent BSA fragment of 50 kDa accumulated in the CTS-triple KO cells (Suppl. Fig. 2C). The slowed degradation of BSA as visualized by the presence of the full-length BSA and the 50 kDa fragment over time was not observed in wild type cells (Suppl. Fig. 2D). In summary, multiple deficiencies of the major lysosomal cathepsin proteases caused accumulation of storage material in lysosomes and a reduced degradation capacity of endocytosed proteins, such as BSA.

### Impaired macroautophagy in multiple cathepsin-deficient cells

**Fig. 4:**
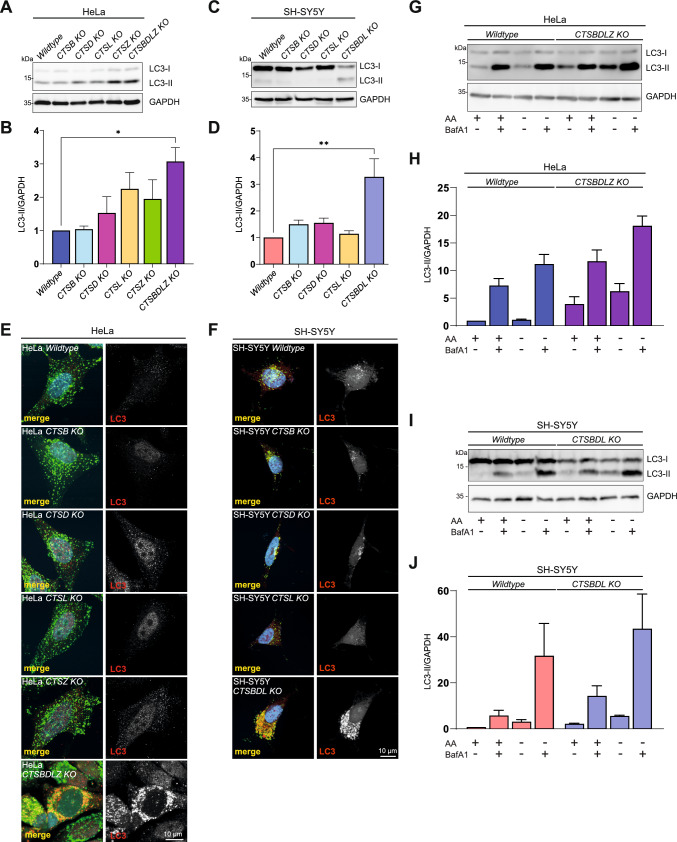
Autophagic flux in the multiple CTS-deficient HeLa and SH-SY5Y cells is impaired. **A** Immunoblot analysis of LC3 in HeLa WT, CTSB, CTSD-, CTSL, CTSZ- and CTSBDLZ-deficient cells. LC3-II levels are increased in the multiple CTS-deficient cells. **B** Quantification of the LC3-II signals reveals significant upregulation if LC3-II only in the HeLa CTSBDLZ-deficient cells. Data represent the mean ± SEM. *p < 0.05. **C** Immunoblot analysis of LC3 in SH-SY5Y WT, CTSB-, CTSD-, CTSL, and CTSBDL-deficient cells. LC3-II levels are increased in the multiple CTS-deficient cells. **D** Quantification of the LC3-II signals reveals significant upregulation if LC3-II only in the SH-SY5Y CTSBDL-deficient cells. Data represent the mean ± SEM. **p < 0.01. Immunostainings of LC3 of HeLa **E** and SH-SY5Y **F** WT, single CTS- and multiple CTS-deficient cells. In both cell lines, HeLa and SH-SY5Y, the multiple CTS KO reveals an accumulation of LC3 in LAMP1-positive vesicles. An autophagic flux assay conducted in **G**, **H** HeLa WT and CTSBDLZ KO cells and in **I**, **J** SH-SY5Y WT and CTSBDL KO cells reveals only a mild impairment in the autophagic flux. Cells were grown in EBSS in nutrient-rich conditions with amino acids (AA) with ( +) or without ( −) the lysosomal inhibitor BafA1 or in EBSS under starvation conditions (− AA) with or without BafA1 for 3 h. *AA* amino acids, *BafA1* Bafilomycin A1, *kDa* kilodalton

Autophagy is a multistep process starting with the formation of a phagophore and later forming the autophagosome, which then fuses with lysosomes to degrade the residing macromolecules, including proteins, by lysosomal proteases for recycling of single amino acids or small peptides [43, 44]. The microtubule-associated protein 1A/1B light chain 3B (MAP1LC3B, short: LC3) is a marker for autophagosomes and is widely used to investigate autophagy [44]. Lysates of HeLa-WT, CTSB, CTSD, CTSL, CTSZ, and CTSBDLZ KO cells, as well as SH-SY5Y WT, CTSB, CTSD, CTSL, and CTSBDL KO cells were investigated by immunoblot for LC3-II levels. LC3-II is a lipidated form of LC3 directly associated with autophagosomal membranes. WT cells and the single KO of CTSB, CTSD, or CTSL in the SH-SY5Y (or CTSB, CTSD, CTSL, CTSZ in Hela) cells did not alter the LC3-II protein levels. However, a combined CTS KO in both HeLa and SH-SY5Y cells led to significant upregulation of LC3-II protein levels (Fig. 4A–D). These findings could be confirmed by microscopy analysis where LC3 and LAMP1 staining revealed prominent and strong staining only in cells devoid of multiple cathepsins but not in wildtype or single-deficient CTS cells (Fig. 4E, F). The elevated levels of LC3-II in the CTSBDL/CTSBDLZ-deficient cells could be caused by either an increase in the formation of autophagosomes (induction of autophagy) or by a blockage in the fusion of autophagosomes with lysosomes (blockage of autophagy) or by both processes. To distinguish these two pathways, an autophagic flux assay was conducted. Cells were either grown under non-starvation (including essential and non-essential amino acids) or starvation conditions with or without Baf A1 for 3 h [44]. Starvation conditions were applied to determine the LC3-II protein accumulation within autophagosomes as autophagy is induced. By adding BafA1 under starvation conditions, the contribution of lysosomal degradation of LC3-II can be revealed [45]. Already under basal conditions, we observed an increased level of LC3-II in the HeLa CTSBDLZ (Fig. 4G, H) and SH-SY5Y CTSBDL-deficient cells (Fig. 4I, J) compared to wildtype cells. However, in both cathepsin-multiple deficient cell lines, it was still possible to additionally induce the formation of LC3-II under starvation and even further by the addition of BafA1. About 35% (HeLa) and 13% (SH-SY5Y) of the autophagic flux is possibly due to the activity of the analyzed lysosomal proteases calculated as the percentage of LC3-II levels under conditions of amino acid starvation as compared to conditions without amino acids and bafilomycin A1 addition in the multiple CTS KO cells. In other words, the majority of the autophagic flux is mediated by the remaining and possibly compensating lysosomal proteases. In control experiments, a still-functioning lysosomal fusion with autophagosomes despite the absence of major cathepsin proteases was observed (Suppl. Fig. 3 A, B) when a tandem mRFP-EGFP-LC3 reporter construct [46] was transfected into HeLa and SH-SY5Y cells, suggesting that the reduced autophagic flux is due to the loss of proteolytic lysosomal activity.

### Cathepsin-multiple deficiency leads to differential protein abundance, as revealed by whole-cell proteome analysis

**Fig. 5:**
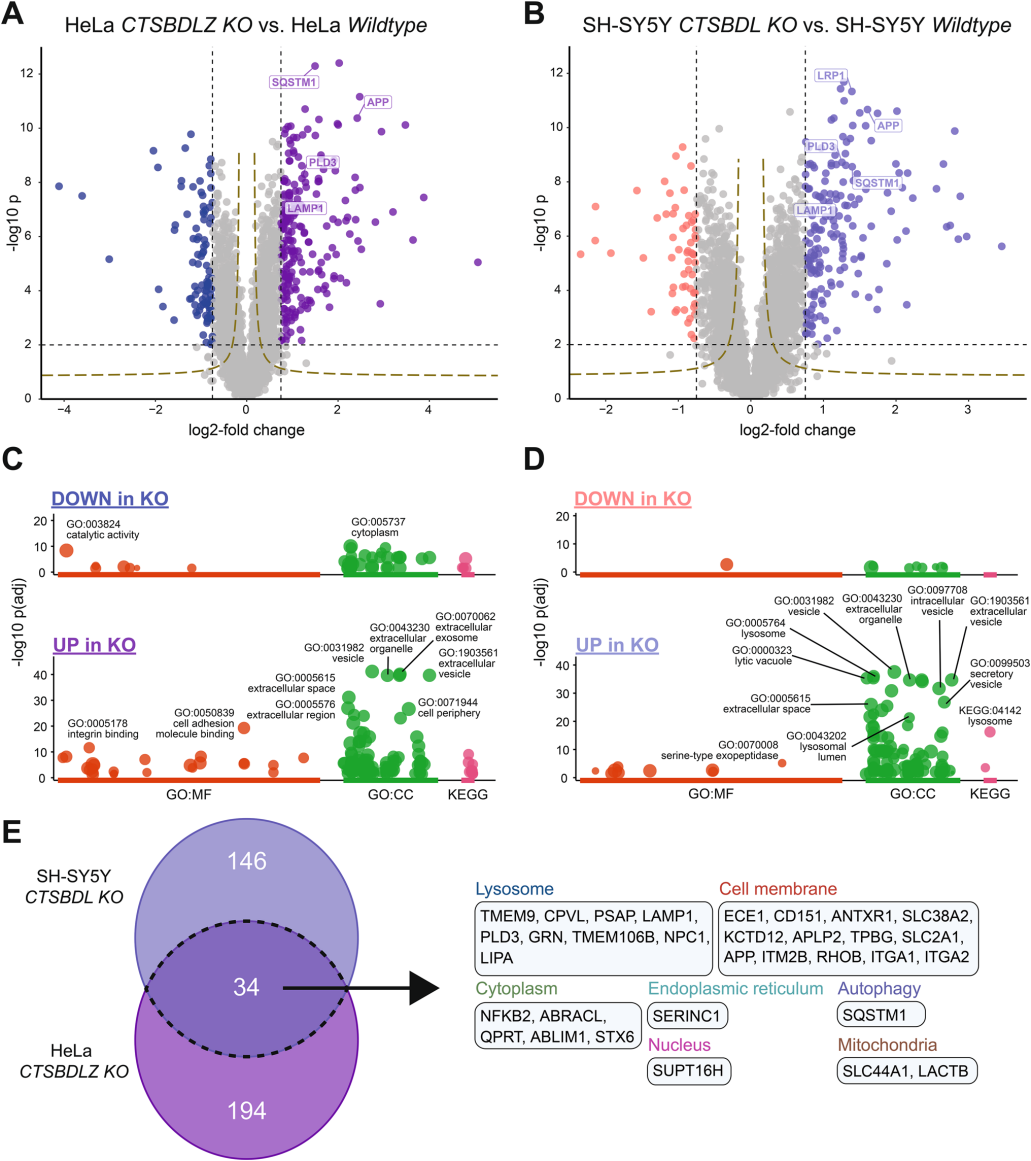
Proteome analysis of HeLa and SH-SY5Y WT and CTSBDL(Z)-deficient cell lines reveals dysregulated protein expression. Volcano scatter plots of **A** HeLa WT vs. CTSBDLZ-deficient (clone 1) cells and **B** SH-SY5Y WT vs. CTSBDL-deficient (clone 9) cells. Select proteins are labelled using their gene name. Black dashed lines indicate p-value (− log10 p = 2.0) and log2-fold change (− 0.75 and 0.75) cut-offs and significantly changed proteins are color-coded as in Fig. 2. We also included hyperbolic curves (golden dashed lines) stemming from the permutation-based FDR correction calculated. **C** Gene ontology (GO) analyses of proteins less (upper panels) or more (lower panels) abundant in CTSBDLZ KO HeLa cells (**C**) and CTSBDL KO SH-SY5Y cells (**D**) when compared to lysates of the corresponding parental cells. Analyses were performed and visualized using the gprofiler2 R package [76]. Select significantly enriched terms are labelled. **E** Venn diagram comparing the sets of proteins significantly more abundant in CTSBDLZ KO HeLa and CTSBDL KO SH-SY5Y cells. The 34 proteins observed as more abundant in both cellular models following cathepsin depletion are listed using their corresponding gene names and grouped based on their annotated subcellular localization

The present data revealed that combined CTS deficiency led to enlarged lysosomes filled with autofluorescent storage material. To identify proteins accumulating within the HeLa CTSBDLZ-KO and SH-SY5Y CTSBDL-KO cells, whole-cell lysates (and not from isolated lysosomes due to the lysosomal storage in the multiple KO cells) were analyzed by bottom-up proteomics. This revealed that the cellular abundance of numerous proteins was significantly changed when CTS-deficient cell lines were compared to the corresponding wildtype cells (Fig. 5A, B). In agreement with our immunoblot data, among the significantly more abundant proteins in the multiple CTS-depleted cells were lysosomal membrane proteins like LAMP1, LAMP2, but also LIMP2 (Lysosomal integral membrane protein 2), and PLD3 (Phospholipase D3), the autophagy marker p62 (Sequestosome-1, SQSTM1), proteins involved in cell adhesion like ITGA1 (Integrin alpha-1) and ITGA2 (Integrin alpha-2) and proteins involved in neurodegenerative diseases like APP (Amyloid Precursor Protein), and LRP1 (Low density lipoprotein receptor-related protein 1). Interestingly, gene ontology (GO) enrichment analyses (Fig. 5C (HeLa), D (SH-SY5Y)) revealed a marked overrepresentation of GO terms relating to extracellular functions, vesicles, and proteins of the cell periphery (HeLa cells). In addition, GO terms related to intracellular vesicles and lysosomes (SH-SY5Y cells) were significantly overrepresented in cathepsin-deficient cells. A direct comparison of the proteome datasets revealed that HeLa and SH-SY5Y multiple cathepsin-deficient cells shared 34 common proteins that were significantly more abundant (Fig. 5E) and a number of proteins that are cell-type-specifically regulated. Interestingly, these are chiefly annotated as proteins localizing to lysosomes or the cell membrane but also include proteins localizing to the ER, cytoplasm, nucleus, or mitochondria. Our analysis suggests that lysosomal cathepsins are cell-type-specific and important factors regulating cellular proteostasis of different protein classes, possibly delivered to lysosomes by means of endocytosis or autophagic pathways.

### Amyloid precursor protein is a prototype protein degraded by multiple lysosomal cathepsin proteases

**Fig. 6:**
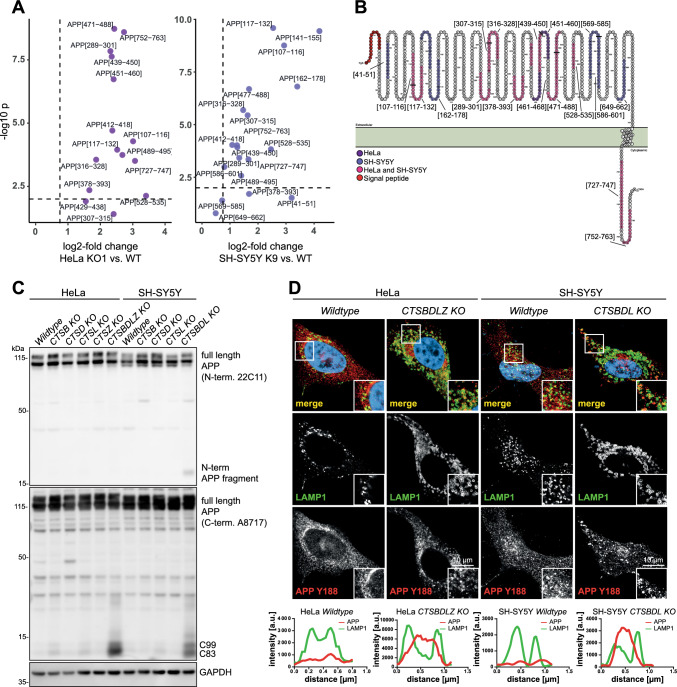
APP accumulates within enlarged lysosomes in multiple CTS-deficient HeLa and SH-SY5Y cells. **A** Volcano plot showing differences in the abundance of tryptic peptides matched to human APP between lysates of CTSBDLZ and parental HeLa cells (left) and CTSBDL and parental SH-SY5Y cells (right). The start and end positions of the matched peptides in relation to APP (770 aa reference isoform) are indicated in square brackets. Dashed lines indicate p-value (− log10 p = 2.0) and log2-fold change (0.75) cut-offs. **B** Visualization of the coverage of APP770 by matched tryptic peptides significantly more abundant in samples of SH-SY5Y CTSBDL KO and HeLa CTSBDLZ KO compared to parental cells. The signal peptide is colored in red, matched and quantified peptides are shown in purple with annotated aa positions. The illustration was generated using Protter [77] and modified subsequently. **C** Immunoblot analysis of APP fragments of HeLa and SH-SY5Y WT, single CTS KO, and multiple CTS KO cells. In SH-SY5Y CTSBDL-deficient cells, a 17 kDa N-terminal APP fragment was detected. In both HeLa and SH-SY5Y multiple CTS-deficient cells, the C-terminal APP fragments APP-C83 and C99 are accumulated, indicating an impaired degradation due to the cathepsins. **D** Immunofluorescence analysis of APP and LAMP1 in HeLa and SH-SY5Y WT and CTSBDL(Z)-deficient cells reveal accumulation of APP in enlarged lysosomes (**C**, **D**)

Interestingly, the proteome analysis revealed APP as one of the common proteins accumulating in the absence of CTS proteases (Fig. 5A, B and E). APP is known to be subject to proteolytic processing mediated by various -including lysosomal-proteases, often with key (patho-)physiological implications [47]. To obtain a deeper insight into APP's lysosomal proteolytic processing, we filtered peptide data for tryptic peptides matched to human APP in CTSBDL-deficient and parental SH-SY5Y cells. Notably, the majority of these peptides are significantly more abundant in the protease-deficient HeLa and SH-SY5Y cells (Fig. 6A). As evident from the visualization in Fig. 6B, these peptides appear to be evenly covering full-length APP without an overt enrichment in known APP cleavage products. We did not observe APP-derived tryptic peptides that were less abundant in cathepsin-deficient cells (Fig. 6A). By immunoblot analysis, both C-terminal APP fragments (C83, C99) accumulated in both quadruple HeLa CTS-BDLZ- and triple SH-SY5Y CTS-BDL-KO cells and an additional ~ 17 kDa N-terminal APP fragment was observed in the SH-SY5Y CTSBDL-deficient cells (Fig. 6C). Importantly such an accumulation was not observed in the single CTS KO cells suggesting a concerted activity of the different CTS proteases in APP degradation. Microscopy analysis of both cell types with antibodies directed against the C-terminus of APP also revealed that APP immunoreactivity was mainly confined to LAMP1-positive lysosomes in the multiple KO cells (Fig. 6D), supporting that lysosomal CTSs are major regulators of APP homeostasis. Next, it was examined whether the altered abundance of certain proteins was only due to the loss of CTSs or if indirect effects (e.g., clonal differences) may have been involved. Therefore, HeLa WT and HeLa CTSBDL-deficient cells (Suppl. Fig. 4A), as well as SH-SY5Y WT and CTSBDL-deficient cells (Suppl. Fig. 4C), were transfected with pcDNA3.1 empty vector and/or pcDNA3.1-CTSB, pcDNA3.1-CTSD, pcDNA3.1-CTSL (and pcDNA3.1-CTSZ for HeLa cells) or all four CTDBDLZ (HeLa) or three CTSBDL (SH-S5Y) encoding plasmids. As evident from the immunoblots, all cathepsins could be successfully re-introduced following transfection with the corresponding expression plasmid. The detection of the mature form of CTSB, CTSD, CTSL (and CTSZ) also demonstrated the successful rescue of the activity of the respective CTSs within the re-transfected HeLa CTSBDLZ or SH-SY5Y CTSBDL (CTSBDL)-deficient cells. Analysis of LAMP1 (Suppl. Fig. 4 B, D) revealed only a trend towards decreased protein expression levels by 40% after re-transfection of CTSB, CTSD and CTSL into the SH-SY5Y CTSBDL-deficient cells and no change in the multiple CTS-deficient HeLa cells. However, the re-expression led to a significant reduction of the ~ 17 kDa N-terminal APP fragment by 66% in the SH-SY5Y CTSBDL KO cells transfected with CTSB, CTSD, and CTSL. The expression of the accumulating C-terminal APP fragments (C88, C99) could also be partially rescued. Finally, the expression of the autophagosome marker LC3-II could be partially rescued after all three CTSs were re-transfected.

These results confirmed that a loss of CTSB, CTSD, and CTSL in SH-SY5Y and CTSB, CTSD, CTSL, and CTSZ in HeLa cells led to dysregulation of certain proteins that had been found by proteome analysis. Also, it revealed that re-transfection of the missing CTSs into the KO cells restored their activity and partially rescued upregulated proteins like APP and LC3-II. Probably, all three cathepsins are required to degrade N-terminal and C-terminal APP fragments, respectively. It should be noted, that the level of full-length APP levels did not appear to be affected in the absence of our disrupted cathepsin proteases which may be explained that it is more subject to processing with a different kinetic along the secretory and early endocytic pathway (e.g. by secretases), whereas after longer periods N- and C-terminal fragments will enter the lysosome where they are degraded by cathepsins.

### Analysis of semi-tryptic peptides reveals a distinctive cleavage pattern in cathepsin-deficient cells

**Fig. 7:**
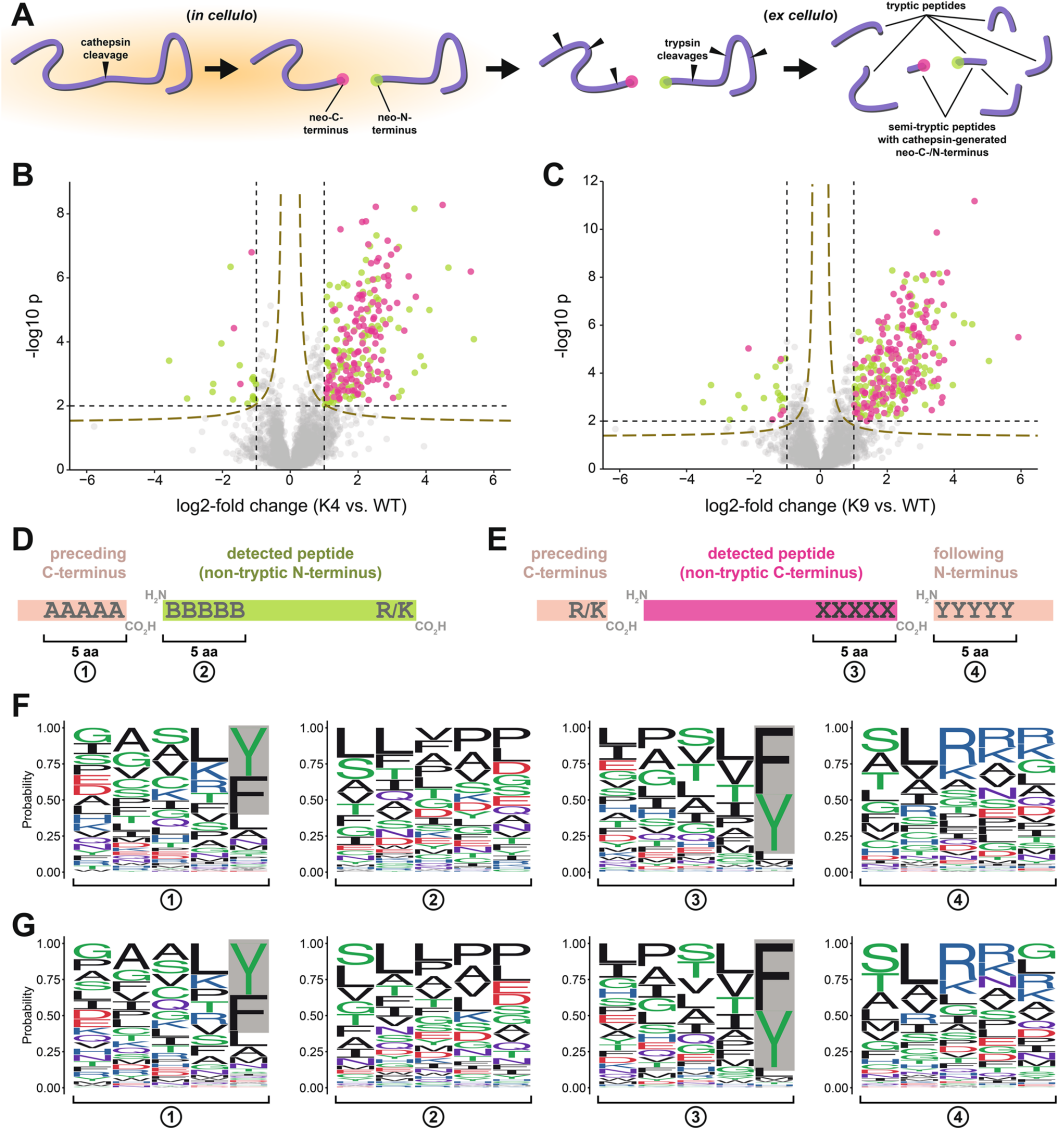
Cleavage site analysis based on semi-tryptic peptides detected in proteome data of cathepsin-deficient SH-SY5Y cells. **A** In living cells, a given substrate (purple chain) can be cleaved by an endopeptidase activity. This results in two fragments derived from the initial substrate that features a neo-C- and a neo-N-terminus, respectively, each situated directly adjacent to the scissile bond cleaved. Following lysis, proteins and protein fragments generated inside live cells are subjected to tryptic digestion in the bottom-up proteomics workflow. This may result in the generation of semi-tryptic peptides that continue to feature the neo-C- or neo-N-terminus originally generated by protease activity inside the cells. **B**, **C** Volcano plots depict changes in the abundance of semi-tryptic peptides between CTSBDL KO clone K4 (**B**) and K9 (**C**) and parental SH-SY5Y cells. Significantly changed semi-tryptic peptides (− log10 p > 2.0 and log2-fold change > 1 or <  − 1) are color-coded as indicated. We also included hyperbolic curves (golden dashed lines) stemming from the permutation-based FDR correction calculated. **D**, **E** Schematic illustration of the cleavage site analysis approach using detected semi-tryptic peptides in the SH-SY5Y datasets. A peptide with a non-tryptic N-terminus (green, **D**) carries an Arg/Lys residue, but in the parent protein, the peptide's most N-terminal amino acid residue is not immediately preceded by an Arg/Lys residue. As this peptide may represent the C-terminal cleavage product of an intracellular cleavage event, the most N-terminal amino acids of the peptide (**2**) but also the amino acids directly preceding its sequence in the parent protein (**1**) may be informative in regards to substrate specificities of proteases active in the cell. Similarly, a peptide with a non-tryptic C-terminus (i.e., not carrying a C-terminal Arg/Lys residue but directly preceded in the parent protein by an Arg/Lys residue) may represent the N-terminal fragment of a proteolytic cleavage event and its C-terminus (**3**) but also the directly ensuing amino acids in the parent protein (**4**) may be informative. **F**, **G** Seqlogo plots showing the probability of individual amino acid residues in motifs 1, 2, 3, and 4 as detailed in **D** and **E** for those semi-tryptic peptides significantly more abundant in CTSBDL KO clone K4 (**F**) and K9 (**G**) than in parental SH-SY5Y cells. The grey background highlights the enrichment of Phe/Tyr residues in the amino acid positions directly C-terminal to a putative intracellular proteolytic cleavage event

There is still limited knowledge about cleavage specificity mediated by cathepsin proteases. Importantly, analysing changes in full-length substrate proteins might only reveal minor insight into their proteolytic capability because smaller peptides derived from partial degradation most likely make up a huge proportion of the substrates of overall proteolytic capacity. For proteomics analyses, proteins are commonly digested using trypsin, which cleaves C-terminally of lysines and arginines to generate peptides well suited for mass spectrometry. Semitryptic peptides have one terminus generated by trypsin but contain non-tryptic N- or C-termini that may stem from cellular protease (e.g., cathepsin) activity (Fig. 7A). Therefore, semi-tryptic peptides can principally be informative in respect to ongoing proteolytic cleavage events in samples. Aiming to get insights into cathepsin protease specificity and ongoing proteolytic processes in cathepsin-deficient cells compared to parental cells, we searched our proteome data for semi-tryptic peptides. For SH-SY5Y cells, the peptide data indeed contained a substantial number of matched and quantified semi-tryptic peptides. As evident from the Volcano plots in Fig. 7B and C, a number of semi-tryptic peptides (both those carrying a non-tryptic N-terminus and those carrying a non-tryptic C-terminus) were significantly more abundant in trypsin-digested lysate samples of two distinct CTSBDL-deficient SH-SY5Y clones.

We next devised an analysis pipeline to extract motifs (5 amino acids in length) flanking the putative proteolytic event that could have given rise to the neo-N- (Fig. 7D) or neo- C-termini (Fig. 7E) of detected semi-tryptic peptides. This was applied to those semi-tryptic peptides significantly more abundant in CTSBDL-deficient SH-SY5Y clones K4 (Fig. 7F) or K9 (Fig. 7G) than in parental SH-SY5Y cells. Extracted amino acid motifs were compiled in Seqlogo plots to visualize amino acid residue enrichments. Analysis of neo-N-termini (i.e., those contained within matched semi-tryptic peptides and those inferred from detected peptides with neo-C-termini) revealed no noteworthy amino acid enrichment. In contrast, in the case of peptides more abundant in either of the two cathepsin-deficient clones, a marked enrichment of the aromatic amino acids Tyr or Phe was apparent at the very C-terminal position (P1 using cleavage site nomenclature) of a putative intracellular cleavage event. This was consistently observed for both experimentally detected but also inferred neo-C-termini.

Thus, exploiting the detected semi-tryptic peptides, our data suggest that cathepsin B, D, and L-deficiency in SH-SY5Y cells is accompanied by a distinctive cellular cleavage pattern with peptides accumulating that have predominantly aromatic amino acids in their C-termini. This may suggest that, in multiple cathepsin-deficient cells, the remaining—or compensatory—degradative capacity is dominated by chymotrypsin-like serine proteases, which prefer substrates with Phe/Try in P1, e.g. cathepsin A, which was not targeted in the cathepsin-depleted cells established in this study.

## Discussion

Lysosomal proteinases are capable of degrading proteins and peptides that enter the compartment by endocytosis, phagocytosis, and autophagy. Deficiency or impairment of cathepsin function is associated with the development of lysosomal storage diseases, neurodegeneration, and cancer [4]. Due to the essential role of CTSs in lysosomal proteolysis, there is a need to understand how CTSs are functionally embedded in the network of lysosomal proteases and protein degradation pathways. This study focused on the major and most abundant CTSs expressed in human neuroblastoma-derived SH-SY5Y and cervical cancer-derived HeLa cells. In the human brain, Hsu et al. determined the protein expression of CTSD with up to 49%, CTSB with up to 32%, and CTSL with up to 9% of the overall CTS expression, pointing out their abundance and their critical functions in neurons and/or glia [48]. Despite their ubiquitous expression, members of the CTS family appear to be delicately coordinated within the lysosomal enzyme pool. This coordination varies across different tissues and cell types.

The analysis of CTSBDL-triple deficient (KO) SH-SY5Y and quadruple CTSBDLZ-HeLa cells generated by CRISPR/Cas9 editing revealed significantly enlarged lysosomes and accumulated autofluorescent lysosomal storage material. This material could likely represent lipofuscin, an aggregated mixture of protein and lipids [41] within lysosomes, and it might well be a result of decreasing proteolytic capacity. A combined deficiency of CTSB, CTSD, and CTSL (CTSZ) in cells has not yet been described to date. Since CTSBDL-triple or CTSBDLZ-quadruple-deficient mice would most likely not be viable or fertile, our cell-based studies complement in vivo studies. Interestingly, pancreas-specific single and double CTS-KO mice with the combinations CTSBD, CTSBL, and CTSDL have been described [11]. After two months of age, CTSBD-deficient mice developed increased numbers and size of cytoplasmic vacuoles in the pancreas likely originating from lysosomal origin [11]. This study also revealed that only a combined loss of CTSB and CTSD in the pancreas alters the lysosomal/autophagic clearance within this tissue.

The lack of the three/four major CTSs in the investigated cell lines similarly caused an impairment of the autophagic flux. The loss of protease activity, enlarged lysosomes, and accumulated storage material are often associated with a reduced autophagic flow [49, 50]. In a previous study, the expression of the autophagy marker LC3-II, which represents a lipidated form of LC3-I associated with autophagosomal membranes throughout the autophagic maturation [51], was not altered in MEFs deficient in CTSB but significantly upregulated in MEFs deficient in CTSL or CTSBL KO [52]. Analysing neural stem cell-derived astrocytes and brain lysates from CTSD-deficient mice also revealed higher LC3-II levels [9, 23]. Treating CTSD-deficient mice with recombinant human (rh)-proCTSB or rh-proCTSL could partially decrease the LC3-II accumulation caused by the deficiency of the aspartyl-protease indicating a partial functional compensation by both cysteine-proteases [23]. SH-SY5Y and HeLa multiple CTS-deficient cells revealed an accumulation of LC3-II upon starvation, and there is an accumulation of autolysosomes. However, CTSB, CTSD, CTSL, and CTSZ seem to not be the only lysosomal hydrolases able to degrade LC3-II and autophagic cargo as its level is even further increased after BafA1 treatment (maximal lysosomal inhibition of proteolytic activity). This indicates that despite the lack of three/four major lysosomal hydrolases, other proteases are still capable of hydrolyzing substrates such as LC3-II. In fact, proteome analysis of CTSBDL-deficient SH-SY5Y cells revealed significant upregulation of the endopeptidase Legumain, the lysosomal exopeptidase Dipeptidyl-peptidase 7 (DPP7), and the exopeptidases Tripeptidyl-peptidase 1 (TPP1) indicating a possible functional compensatory effect for the loss of the three major lysosomal hydrolases. Analysing the additional contribution of these and other proteases will be an interesting subject for our future studies. An endocytic uptake of secreted lysosomal proteases may also contribute to lysosomal proteolysis. Our findings suggest a high degree of plasticity and functional adaption. However, compensatory adaption reaches limitations if too much proteolytic activity is lost. It is also interesting to note that the current study using well-defined genetic models with a single and multiple lack of CTS proteases possibly allows clearer conclusions as compared to studies using pharmacological approaches such as inhibition of cathepsins with leupeptin/E64, pepstatin or bafilomycin, all known to cause side-effects ranging from affecting cellular trafficking [53] to autophagy and apoptotic pathways [54].

Endocytosed serum albumin was degraded more slowly in the multiple KO cells. To also obtain insight into the substrate usage of the different CTSs, proteome analysis of whole-cell lysates from wildtype and CTS-depleted cells was performed and revealed accumulating proteins in cell lysates, including APP and APP N- and C-terminal fragments. APP is not necessarily a new substrate for CTSB, CTSD, and CTSL [55–57], but the accumulation of a 17 kDa N-terminal APP fragment in multiple CTS-deficient cells might be of specific interest. This fragment is possibly linked to the N-terminal part of APP, which is released as soluble APP (e.g., sAPPα and sAPPβ) into the cytosol [58]. Soluble APPα (sAPPα) and sAPPβ are internalized by endocytosis and delivered to lysosomes by receptor-mediated transport, mainly through LRP1 [59]. Interestingly, LRP1 was also among the upregulated proteins found in proteome analysis of the CTS-deficient cells, and the soluble APP fragments could be better transported to lysosomes. In case the major cathepsins are missing, an N-terminal fragment of APP accumulates. In SH-SY5Y cells, this fragment can be partially degraded after re-transfection of CTSB, CTSD, or CTSL, but the activity of all three cathepsins was required to completely hydrolyze and reduce the appearance of this N-terminal polypeptide. This again indicates a hierarchical and precise interplay of CTSB, CTSD, and CTSL within the lysosomal network.

Previously, it could already be shown that a deficiency of CTSB, CTSL, or both led to the accumulation of C-terminal APP fragments (CTFs) C83 and C99 in MEFs [52, 60–62]. During this study, APP CTFs were only upregulated in the CTSBDL-deficient SH-SY5Y cells but not in any of the single CTS KO cells, providing evidence for functional redundancy and that only the loss of more than one CTS leads to an accumulation of the APP CTFs. Lysosomal cathepsins are involved in the degradation of APP CTFs through autophagy [62]. Conversely, the loss of three major CTSs should lead to an accumulation of these fragments, causing a disturbed degradation. By re-addition of single cathepsins to the SH-SY5Y CTSBDL-deficient cells, the expression of both APP CTFs remained high, i.e., no prominent reduction of the level of these fragments was observed. However, re-transfection of all three cathepsins in parallel led to lowered APP CTF protein levels, suggesting that the APP CTF increase in CTSBDL-deficient SH-SY5Y cells was rescuable. The major degradation of the C83 and C99 APP fragments is indeed mediated by the common activities of CTSs.

Our approach to specifically examine the nature of semi-tryptic peptides (containing neo-N- and C-termini from endogenous proteases) in our proteome datasets was motivated by our interest in learning more about substrate specificity and endogenous CTS-mediated cleavage patterns. While some (semi-tryptic) peptides might be changed as a secondary response to the lysosomal changes in the total cell lysates, a great number likely stem from impaired cathepsin-mediated proteolysis. The combined deficiency of the three major cathepsins (L/B/D) in SH-SY5Y cells led to an accumulation of a characteristic set of semi-tryptic peptides stemming from cleavage events with aromatic residues such as phenylalanine or tyrosin it the P1 position. It seems likely that chymotrypsin-like serine proteases become more important when major endopeptidases are missing, such as in our cellular models. Here, the lysosomal protease cathepsin A, which has a preference for hydrophobic amino acids at the P1 position and is usually assayed by cleavage of synthetic Phe-Leu peptides, may be a prime candidate to exert this activity [63, 64]. CTSA mRNA is also an abundant abundantly expressed in the in the SH-SY5Y cells (Fig. 1A). Functionally, lysosomal CTSA is part of a multienzyme complex, and it is required to stabilize β-galactosidase and activate neuraminidase 1 [65] and mutations in the *CTSA* gene have been linked with galactosialidosis [66]. So far, CTSA substrates have mostly been described as peptide hormone substrates [67], but our data suggest that it might also contribute to lysosomal bulk proteolysis and compensate for the loss activity of other cathepsins as observed in our cellular models.

In summary, our data suggest a need for the concerted but also regulated activities of lysosomal proteinases in membrane protein degradation, in the degradation of endocytosed proteins, and in the regulation of the autophagic flow. In future studies, it will be interesting to elucidate the peptidome of such cathepsin-depleted cells as well as their contribution to the metabolic control mediated by lysosomal proteolysis.

## Material and methods

### Cell culture and Generation of CTS-deficient cells

HeLa and non-differentiated SH-SY5Y were maintained in Dulbecco's modified Eagle Medium (DMEM) containing 4.5 g/L of D-Glucose and L-glutamine (Thermo Fisher Scientific) and were supplemented with 10% (v/v) fetal bovine serum (FBS) and 1% Penicillin/Streptomycin. Cells were cultivated in 5% CO_2_ at 37 °C.

Generation of the different cathepsin-deficient HeLa and SH-SY5Y cell lines was performed using the Neon™ Transfection System (Thermo Fisher Scientific). GuideRNA (guideRNA: CTSB: UGCAGGCAGCUUCAGGUCCU; CTSD: UGGACGUGAACUUGUGCAGC; CTSL: GAUGAUUGAACUGCACAAUC; CTSZ: GGACAGGUACUCAUGAGGCC) Synthego (Redwood City, CA, USA) and Cas9 (IDT) was introduced to the cells according to the manufacturers protocol and recommendations. The next day, the medium was exchanged, and cells were separated into single cells and 96-well plates after they reached a confluency of 90%. Single clones were expanded for sequencing and western blot analysis. The process of generating SH-SY5Y CTSBDL knockout clones was started with a confirmed CTSB-deficient clone, and the guideRNAs for CTSD and CTSL were introduced simultaneously. In HeLa cells CTSL,D and B were first deleted and the triple KO clone used for additional targeting of CTSZ. To validate the single clones, DNA was extracted using DirectPCR® Lysis Reagent Tail (Peqlab) containing 0.3 mg/ml Proteinase K (55 °C overnight, 85 °C for 15 min). After centrifugation, the lysate was used as a template to perform PCR using special sequencing primers covering the target sequence of the guideRNAs. The PCR products were cleaned up using the High Pure PCR Purification Kit (Roche) and sent for sequencing (Eurofins Genomics). Efficiency and genotype editing were analyzed using the Synthego ICE Analysis Tool and TIDE Sequence Analysis Tool. In addition, independent single and multiple CTS-depleted clones were analyzed by western blot analysis to confirm the knockout and the increased LAMP1 expression in different multiple KO clones biochemically.

### Western blot analysis

Cells were washed in ice-cold PBS centrifuged at 500 g for 10 min at 4 °C, harvested with cell scrapers, and lysed in RIPA (50 mM Tris–Cl, 150 mM NaCl, 1% NP-40, 0.05% sodium deoxycholate, 0.01% SDS, pH 7.5) containing 1X protease inhibitor cocktail (Complete) by incubation on ice for 1 h. Protein lysate was collected after centrifugation at 16.000 rpm for 10 min at 4 °C, and concentration was determined using the Pierce BCA Assay (Thermo Fisher Scientific). Lysates were denatured by adding 5X Laemmli Buffer (60 mM Tris–Cl pH 6.8, 2% SDS, 10% glycerol, 5% β-mercaptoethanol, 0.01% bromophenol blue) in a 1:5 ratio and boiled at 95 °C for 5 min. Samples were loaded on a 12.5% polyacrylamide gel or 4–12% Bis–Tris-Gels (NuPAGE, Thermo Fisher Scientific) and exposed to constant voltage of 80 V in 1X SDS Running Buffer (25 mM Tris, 1.5% glycine, 0.1% SDS) or 1X MES Buffer (Thermo Fisher Scientific). Proteins were transferred onto a nitrocellulose membrane (GE Healthcare) using a semi-dry system for 2 h at 65 mA per blot or a tank blot system for 2 h and 250 mA. After the transfer, the membrane was blocked in either 5% milk in TBS-T (2.5 mM Tris, 13.7 mM NaCl, 27 µM KCl, pH 7.4, 1% Tween) for 30 min followed by incubation with the first antibody at 4 °C over night (CTSB (AF953, R&D Systems), CTSD (AF1014, R&D Systems), CTSL (AF952, R&D Systems), CTSZ (AF934, R&D Systems), LAMP1 (1D4B, DSHB), LC3 (PM036, MBL), GAPDH (sc-25778, Santa Cruz Biotechnology), C-terminal APP (A8717, Sigma Aldrich), N-terminal APP (22C11, Sigma Aldrich). Secondary antibodies conjugated to horseradish peroxidase (HRP) (rabbit, mouse, goat) were incubated for 1 h at room temperature in blocking solution. Protein detection was done using ECL and the Amersham Typhoon Imager (GE Lifesciences).

### Immunofluorescence staining

Cells were grown on coverslips, washed in ice-cold PBS, and fixed in 4% paraformaldehyde (PFA) for 20 min, permeabilized with PBS and 0.2% saponin for 5 min, and quenched with PBS, 0.2% saponin and 0.12% glycine for 10 min. Blocking solution (PBS, 0.2% saponin, 10% FBS) was added for 1 h, and then cells were incubated in the first antibody diluted in blocking solution (LAMP1 (1:300), LIMP2 (1:300, L2T2, Pineda Antibody Service, [68]), LC3 (1:300), APP (1.300)) overnight at 4 °C. To detect proteins of interest, cells were then incubated with fluorescence-tagged secondary antibodies (donkey-anti-mouse, donkey-anti-rabbit, donkey-anti-goat) in blocking solution. Cells were mounted on slides with DABCO/Mowiol containing 1 µl/ml DAPI. Imaging was done by using the confocal laser scanning microscope LSM980 Airyscan2 (Zeiss) under equal settings per experiment. Digital images were processed and adjusted equally by ZEN software (Zeiss) or ImageJ (Fiji). Lysosomal size was determined by using the Line-Tool in Zen software to determine the diameter in biological triplicates.

### Autophagic flux and starvation assay

To determine the autophagic flux HeLa WT and BDLZ KO cells and SH-SY5Y WT and BDL KO cells were transfected with a mRFP-GFP fluorescence-tagged LC3 coding plasmid. 1 × 10^5^ HeLa WT and BDLZ and 3 × 10^5^ SH-SY5Y WT and BDL cells were seeded in 6-well plates with a coverslip 24 h prior to the assay being applied. 1 µg mRFP-GFP fluorescence-tagged LC3 or pcDNA3.1-Hygro( +) and Turbofect (Thermo Fisher Scientific) were added to serum-free medium and incubated at room temperature for 15 min prior to the application to cells. After 5 h the medium was exchanged, and cells were incubated for 48 h and used for subsequent experiments. A starvation assay was performed on both HeLa and SH-SY5Y cells. For each condition, 1.5 × 10^6^ HeLa cells and 1.5 × 10^6^ SH-SY5Y cells were seeded on 100 mm plates. The next day, cells were washed in ice-cold PBS three times to remove leftover FBS. Cells were incubated either with EBSS (Thermo Fisher Scientific), 1X essential amino acids (Thermo Fisher Scientific), 1X non-essential amino acids (Thermo Fisher Scientific), and 10% FBS either with or without 500 nM Bafilomycin A1 (Sigma) and EBSS only with or without 500 nM Bafilomycin A1. After 2 h (HeLa) and 3 h (SH-SY5Y) of incubation, cells were washed in PBS and harvested using cell scrapers. Cells were collected by centrifugation at 600 g for 10 min and lysed in RIPA containing 1X Complete, 20 mM NaF, 4 mM glycerol 2-phosphate, and 4 mM NaVO_3_. Lysate collection and protein concentration determination were performed as described.

### BMV-109 labeling

To analyze the activity of the different cathepsin-knockout cell lines, we used the pan-reactive cysteine quenched activity-based-probe (qABP) BMV-109 (provided by M. Bogyo, Stanford, USA). 1.5 × 10^6^ HeLa and 1.5 × 10^6^ SH-SY5Y cells were seeded in 60 mm plates 24 h prior to the assay was applied. Cells were washed in PBS. Depending on the assay, HeLa WT and CTSBDLZ cells and SH-SY5Y WT and CTSBDL KO cells were incubated with 150 µM E64-D (Enzo) in DMEM only for 15 min as controls prior to the application of 100 nM BMV-10 for another 2 h. Cells were harvested and lysed as described while keeping the samples in the dark to prevent bleaching. SDS-PAGE was performed on a 15% polyacrylamide gel and kept in the dark as well. Using the Amersham™ Typhoon™ Biomolecular Imager (GE Healthcare), the in-gel Cy5-fluorescence was detected, and coomassie blue staining (GelCode™ Blue Stain Reagent, Thermo Fisher Scientific) was used as a loading control.

### Electron microscopy

HeLa WT and BDLZ cells and SH-SY5Y WT and BDL cells were washed in PBS and fixated in double-strength fixative (4% paraformaldehyde (PFA), 2% glutaraldehyde (GA) in PBS) for 15 min. Double-strength fixative was discarded and replaced with fixative (4% PFA and 1% GA in PBS). Cells were harvested using a cell scraper and centrifuged for 10 min. The supernatant was discarded. The resulting pellets were shipped in fixative to Dr. M Schweitzer (HH, UKE). The cells were washed in PBS three times and centrifuged at 1000 g for 5 min each time. The resulting pellets were rinsed in 0.1 M sodium cacodylate buffer (pH 7.2–7.4), embedded in 2% agarose, and osmicated using 1% osmium tetroxide in cacodylate buffer. Cells were dehydrated using ascending ethyl alcohol concentration steps and rinsed in propylene oxide two times. Infiltration of the embedding medium was performed by immersing the samples in a 1:1 mixture of propylene oxide and Epon and finally in neat Epon and polymerized at 60 °C. Semithin sections (0.5 μm) from the SH-SY5Y WT and CTSBDL-deficient cells were prepared for light microscopy mounted on glass slides and stained for 1 min with 1% Toluidine blue. Ultrathin sections (60 nm) were examined in an EM902 (Zeiss, Germany). Pictures were taken with a MegaViewIII digital camera (A. Tröndle, Moorenweis, Germany).

### Proteome analysis

HeLa WT and CTS BDLZ-deficient, as well as SH-SY5Y WT and CTS BDL-deficient cell clones (each n = 6), were seeded 24 h after cell pellet collection. Cells were washed in ice-cold PBS and harvested with a cell scraper. A modified RIPA lysis buffer (1% Triton X-100, 0.5% sodium deoxycholate, 0.1% SDS, 150 mM NaCl, 5 mM EDTA, 50 mM TrisHCl pH 8) was used to lyse the cells one ice for 30 min. The supernatant was collected after centrifugation at 16.000 rpm for 10 min at 4 °C. 10 μl was used to determine protein concentration using a BCA assay. Per sample, 25 μg protein was subjected to the filter-aided sample preparation protocol (FASP) [69] using Vivacon spin filters with a 30 kDa cut-off (Sartorius). Proteins were reduced with 20 mM DTT followed by alkylation of cysteine residues with 50 mM iodoacetamide (Sigma Aldrich). Next, proteins were washed with 8 M urea (Sigma Aldrich) and digested first with 0.5 μg LysC (Promega) for 16 h at 37 °C and second with 0.25 μg trypsin (Promega) for 4 h at 37 °C. Generated peptides were eluted by centrifugation and acidified with 8% (v/v) formic acid to pH < 3. Proteolytic peptides were desalted by stop-and-go extraction (STAGE) with self-packed C18 tips (Empore) and dried by vacuum centrifugation [70]. Peptides were dissolved in 20 μl 0.1% formic acid (FA), and protein concentration was determined by a nanodrop photometer at 280 nm.

Peptides were analyzed for label-free protein quantification (LFQ) on an Easy nLC 1200 nanoHPLC (Thermo Scientific), which was coupled online to a Nanospray Flex Ion Source (Thermo Scientific) equipped with a PRSO-V1 column oven (Sonation) to a Q-Exactive HF mass spectrometer (Thermo Scientific). 1.3 μg of peptides were separated on in-house packed C18 columns (30 cm × 75 μm ID, ReproSil-Pur 120 C18-AQ, 1.9 μm, Dr. Maisch GmbH) using a binary gradient of water (A) and acetonitrile (B) supplemented with 0.1% formic acid (0 min, 2% B; 3:30 min, 5% B; 137:30 min, 25% B; 168:30 min, 35% B; 182:30 min, 60% B) at 50 °C column temperature.

Data-dependent acquisition (DDA) was used for LFQ. Full MS scans were acquired at a resolution of 120,000 (m/z range: 300–1400; automatic gain control (AGC) target: 3E + 6). The 15 most intense peptide ions per full MS scan were selected for peptide fragmentation (resolution: 15,000; isolation width: 1.6 m/z; AGC target: 1E + 5; normalized collision energy (NCE): 26%). A dynamic exclusion of 120 s was used for peptide fragmentation.

The MS raw data was analyzed with the software Maxquant, version 1.6.15.0 (maxquant.org, Max-Planck Institute Munich) [71]. The MS data was searched against a reviewed canonical fasta database of Homo sapiens from UniProt (download: 2020-01-16, 20617 entries). Trypsin was defined as a protease. For semitryptic peptide searches, the same settings were used in Maxquant 1.6.15.0 but allowed for semispecific free peptide N- and C-termini. Two missed cleavages were allowed for the database search. The option first search was used to recalibrate the peptide masses within a window of 20 ppm. For the main search peptide and peptide fragments, mass tolerances were set to 4.5 and 20 ppm, respectively. Carbamidomethylation of cysteine was defined as a static modification. Acetylation of the protein N-terminal and oxidation of methionine were set as variable modifications. The false discovery rate for both peptides and proteins was adjusted to less than 1%. The “match between runs” option was enabled with a matching window of 1 min. LFQ of proteins required at least two ratio counts of unique peptides. The protein LFQ reports of Maxquant were further processed in Perseus version 1.6.14 [72]. The protein LFQ intensities were log2 transformed, and log2 fold changes were calculated between cathepsin KO clones and respective WT controls. A Student’s t-test with a two-tailed distribution was applied to evaluate the significance of proteins with changed abundance. For semitryptic peptides, peptide LFQ intensities were used in a manner similar to the protein LFQ data analysis. A permutation based false discovery rate correction (p = 0.05; s0 = 0.1) was applied to account for multiple comparisons [73].

Volcano plots were generated using the ggplot2 package, and gene ontology analyses were performed using the gprofiler2 package (with default settings) in R. For the cleavage site analysis, peptides significantly more abundant in cathepsin-deficient SH-SY5Y cells and featuring a non-tryptic N-terminus on the one hand or C-terminus, on the other hand, were extracted from the list of identified semi-tryptic peptides. In R, non-tryptic N- or C-terminal amino acid motifs were extracted (length = 5 aa). Using full protein sequences deposited in Uniprot, the five amino acids preceding the very N-terminus of peptides with a non-tryptic N-terminus and the five amino acids following the very C-terminal residue of peptides with a non-tryptic C-terminus were extracted. Duplicate amino acid motifs as well as motifs shorter than 5 amino acids, were removed. The amino acid distribution in the individual positions was then analyzed and visualized using the ggseqlogo package in R [74].

### Transient transfection

Cells were seeded on a cell culture plate (1–1.5 × 106 cells for a 6 cm dish) with a cover slip if needed for a following experiment one day prior to transfection. The next day, 1–3 μg of the desired plasmid was incubated with Turbofect in 300 μl DMEM for 15 min at RT. Then, the solution was added to the cells, and after 5–8 h, the medium was changed. Cells were grown for 48 h until further usage.

### Endocytosis and degradation analysis of BSA-647

Bovine serum albumin (BSA) is a broad substrate for proteases and a known substrate of CTSD. Therefore, it can be used to examine endocytosis, its intracellular processing, and analysis of enzymatic activity of proteins hydrolyzing BSA. 1.5 × 10^6^ SH-SY5Y WT and CTSBDL-deficient cells were seeded on 6 cm cell culture plates 24 h prior to the assay was conducted. Cells were washed in PBS, and 15 μg/ml BSA-Alexa 647 in DMEM containing 1% FBS and 1% P/S was added to the cells. Two types of experiments were performed using this method. First, cells were incubated with BSA-Alexa 647 for 1, 3, 5, 6, 8, 12, 14 and 16 h and harvested afterward. Second, cells were incubated with BSA-Alexa 647 for 2 h, then washed with PBS and incubated for another 10, 20, 30 min, 1 h, and 2 h in DMEM containing 1% FBS and 1% P/S (pulse-chase experiment). Cells were harvested and lysed under the protection of light. A fluorescent SDS-PAGE was used to analyze the samples. The in-gel fluorescence signal of BSA-Alexa 647 was detected using the Amersham™ Typhoon™ Biomolecular Imager (GE Healthcare).

### DQ-BSA-647 cleavage assay

CTS-mediated BSA cleavage was determined using HeLa WT, HeLa CTSBDLZ KO, SH-SY5Y, and SH-SY5Y BDL KO cells. All cells were seeded 24 h before the experiment. Before the assay, the cell medium was removed, and all cells were washed 1 × with DMEM w/o phenol red and subsequently incubated in DMEM w/o phenol red and 1% FCS. HeLa WT and HeLa CTSBLDZ KO cells were incubated with 100 µg, and SH-SY5Y and SY-SY5Y BDL KO cells were incubated with 25 µg of DQ-BSA647 for four hours, in the presence or absence of BafA1 (250 nM) before live cell imaging performed.

### Statistical analysis

The detection of signal intensities in immunoblots and immunofluorescence stainings was performed with the software ImageJ. Statistical analysis was done with GraphPad Prism 8. Statistics were generated using two-tailed students *t*-test to analyse wildtype against respective knockout cells. Among groups, statistics were carried out using one-way ANOVA and Bonferroni multiple comparison post hoc test. *P*-values < 0.05*, 0.0021**, 0.0002***, and 0.0001****

### Supplementary Information

Below is the link to the electronic supplementary material.

Supplementary file1 (PDF 7708 KB)

## Data Availability

The mass spectrometry proteomics data have been deposited to the ProteomeXchange Consortium via the PRIDE (Perez-Riverol et al. 2022)) partner repository with the dataset identifier PXD048603. Reviewer account details: Login at https://www.ebi.ac.uk/pride/login; Username: reviewer_pxd048603@ebi.ac.uk. Password: YMpJh2eS. Perez-Riverol Y, Bai J, Bandla C, Hewapathirana S, García-Seisdedos D, Kamatchinathan S, Kundu D, Prakash A, Frericks-Zipper A, Eisenacher M, Walzer M, Wang S, Brazma A, Vizcaíno JA (2022). The PRIDE database resources in 2022: A Hub for mass spectrometry-based proteomics evidences. Nucleic Acids Res 50(D1):D543-D552.
