# Supplementary material for: Cellular depletion of major cathepsin proteases reveals their concerted activities for lysosomal proteolysis
Source: Cell Mol Life Sci. 2024 May 22;81(1):227. doi: 10.1007/s00018-024-05274-4 (PMC11111660; doi:10.1007/s00018-024-05274-4)
Supplement: Supplementary file 1 — Supplementary file1 (PDF 7708 KB) [file 18_2024_5274_MOESM1_ESM.pdf]

Supplement Figure 1

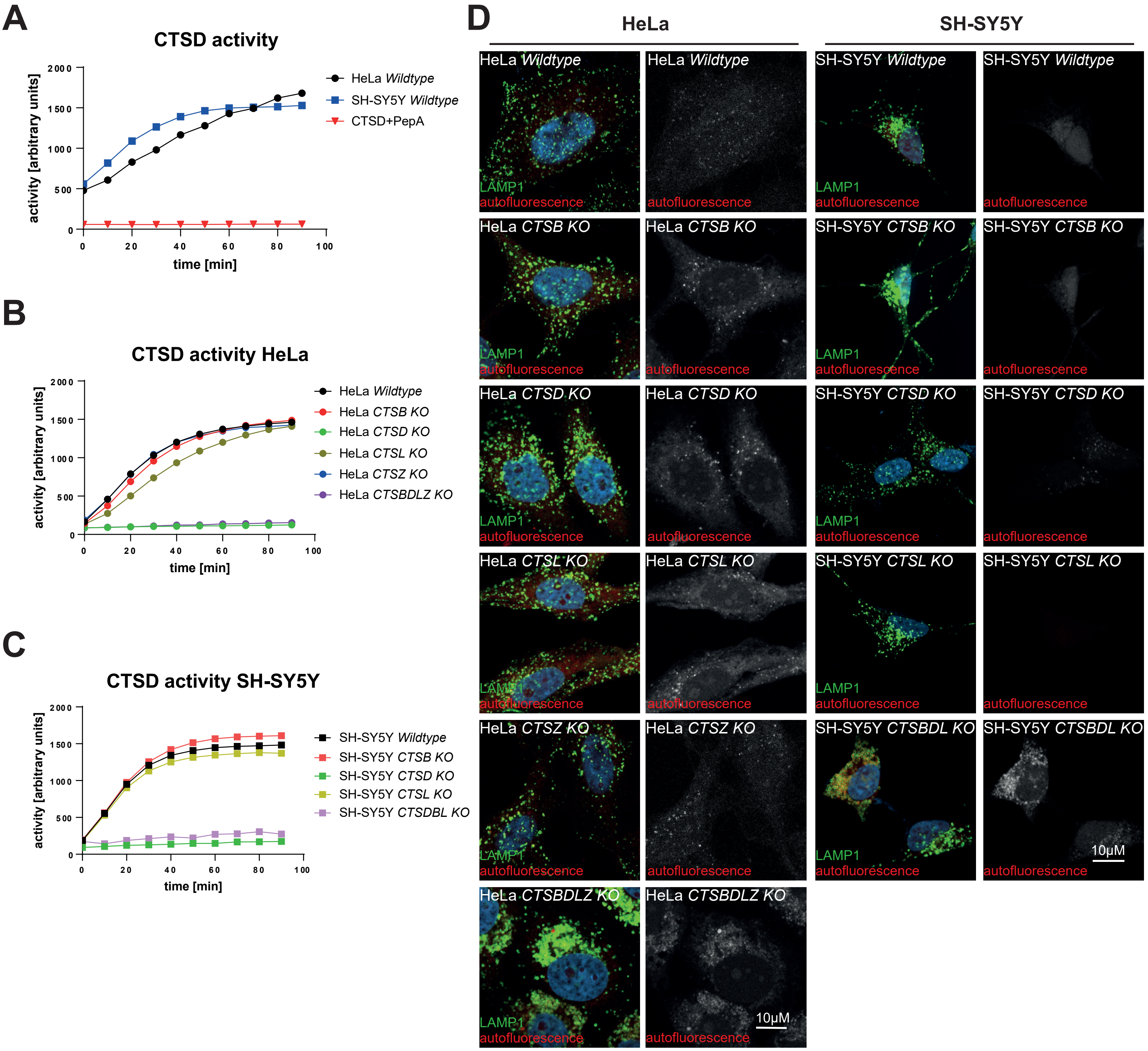

Supplementary Figure 1. Activity of the aspartyl-type protease CTSD and autofluorescence analysis in HeLa and SH-SY5Y cells. (A) Measured CTSD-activity per µg protein over time. Cleaved CTSD-substrate fluorescence intensity measured after excitation at 360 nm at 440nm. CTSD-activity is shown for HeLa, and SH-SY5Y WT cells, and pre-activated rh-CTSD co-incubated with Pepstatin A (PepA). (B) CTSD activity was measured in HeLa WT, CTSB, CTSD, CTSL, CTSZ, and CTSBDLZ KO cells. (C) CTSD activity measured in SH-SY5Y WT, CTSB, CTSD, CTSL, and CTSBDL

KO cells. (D) SH-SY5Y and HeLa wildtype and single CTS-deficient cells stained with LAMP1 (green at 488nm). Stainings were analyzed at the excitation wavelength of 594 nm to detect autofluorescent signals of lipofuscin (autofluorescence, red). Scale bar: 10 μM.

Supplement Figure 2

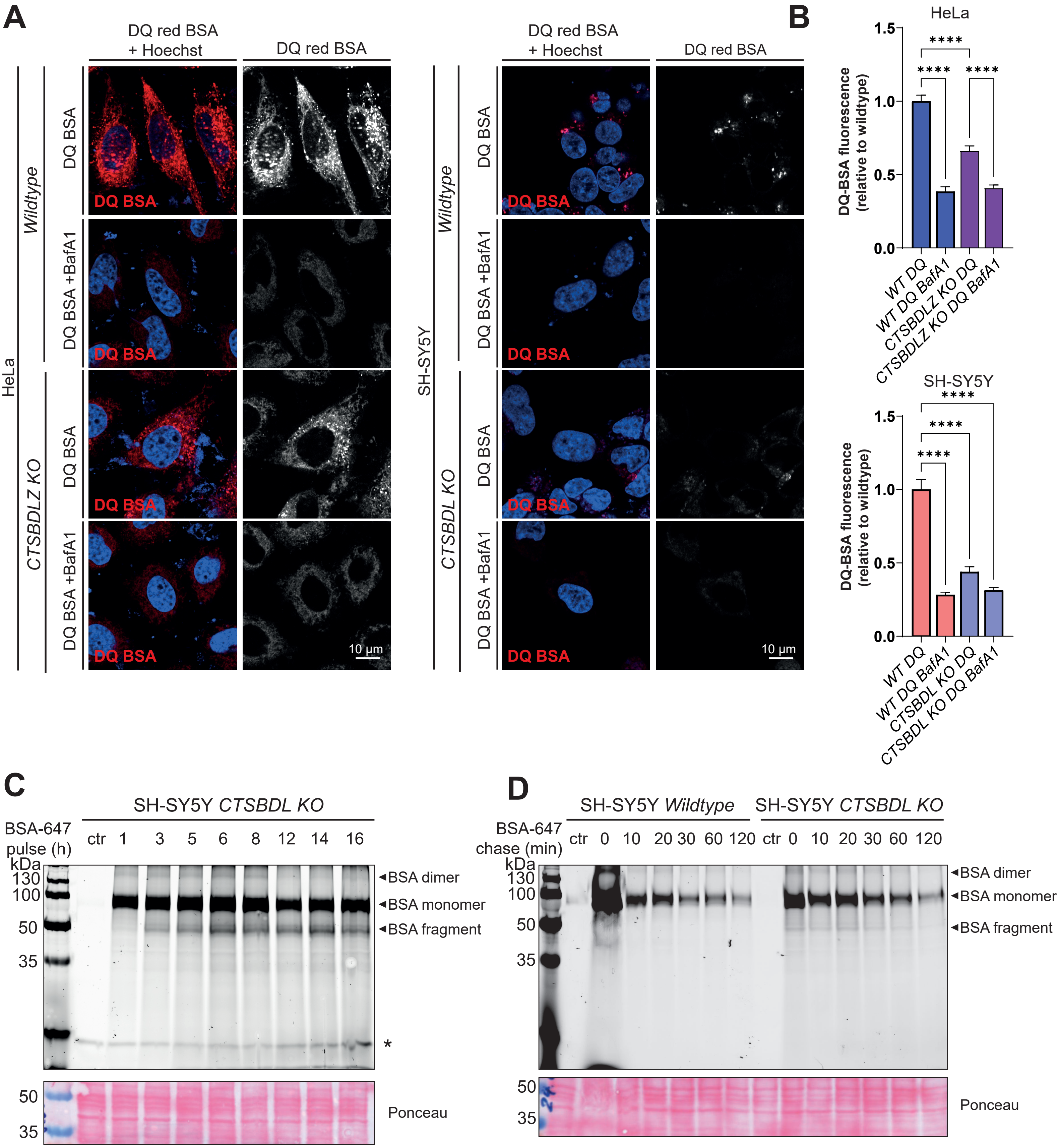

Supplementary Figure 2. Reduced capacity to degrade endocytosed material. (A) Images of the uptake of DQ-red BSA and (B) quantitative analysis of fluorescent signal of HeLa WT and CTSBDLZ-deficient and SH-SY5Y WT and CTSBDL-deficient cells. HeLa WT cells show more DQ-red BSA signal overall compared to SH-SY5Y cells. The DQ-red BSA fluorescent signal is significantly reduced in both multiple CTS-deficient cell lines compared to their respective WT. Treatment with Bafilomycin A1 (BafA1) in both cell types, HeLa and SH-SY5Y, leads to complete inhibition in the degradation of BSA, resulting in low or almost no fluorescent signal. Data represent the mean ± SEM. **** p<0.0001. Scale bar: 10 µm. (C) The in-gel signal intensities of BSA-Alexa647 reveal that endocytosis of BSA is not impaired in the SH-

SY5Y CTSBDL-deficient cells. (D) SH-SY5Y WT and CTSBDL-deficient cells were incubated with 15 µg/ml BSA Alexa Fluor 647 conjugate for 2 h (pulse) and chased for 0, 10, 20, 30, 60 and 120 min. Controls were harvested 120 min after pulsing cells without the BSA conjugate. The in-gel fluorescent signal of BSA-Alexa 647 shows the accumulation of a ~50 kDa BSA fragment in the SH-SY5Y CTSBDL-deficient cells seems to be degraded with time. Ponceau S staining afterwards served as a loading control. kDa: kilodalton.

Supplement Figure 3

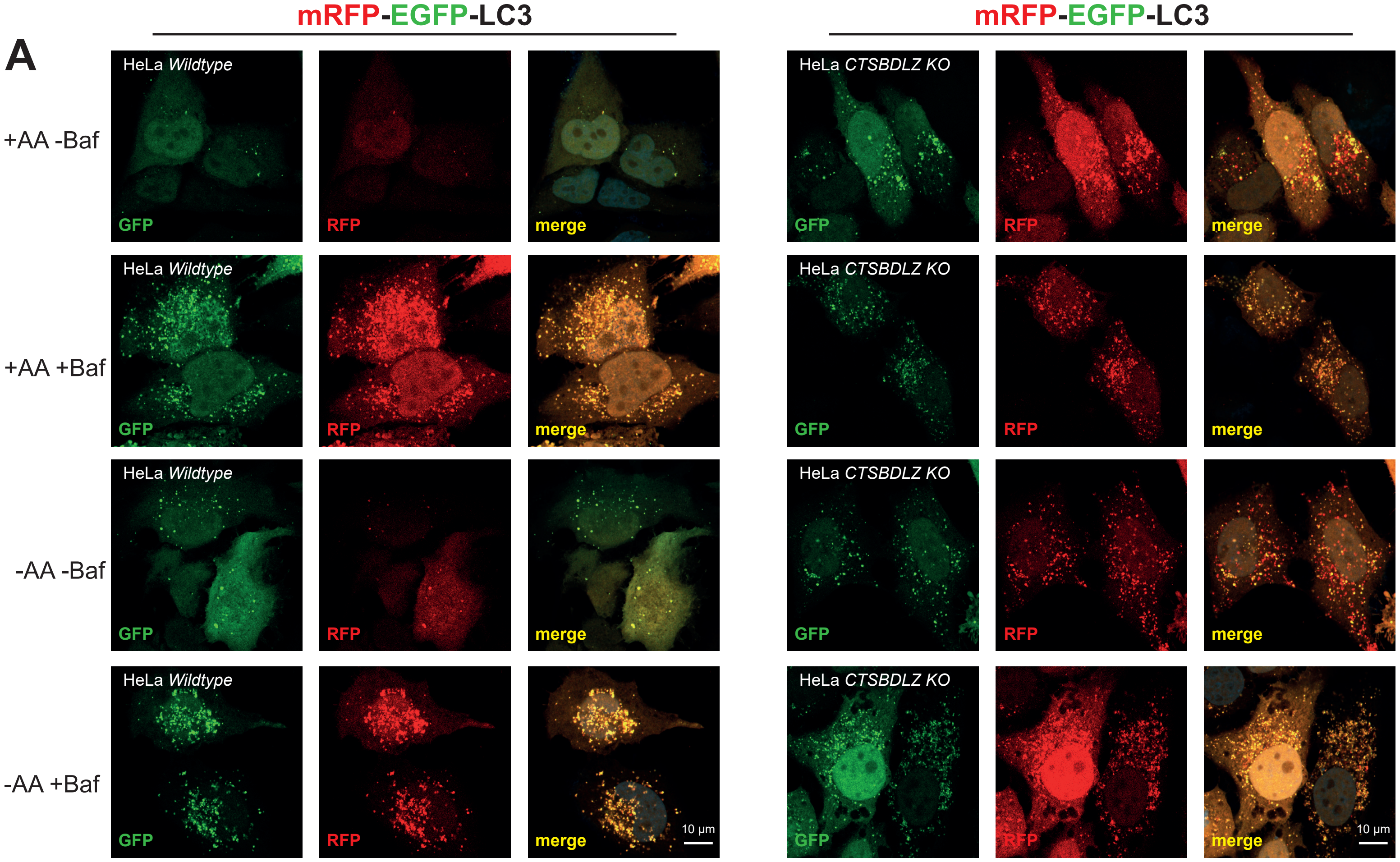

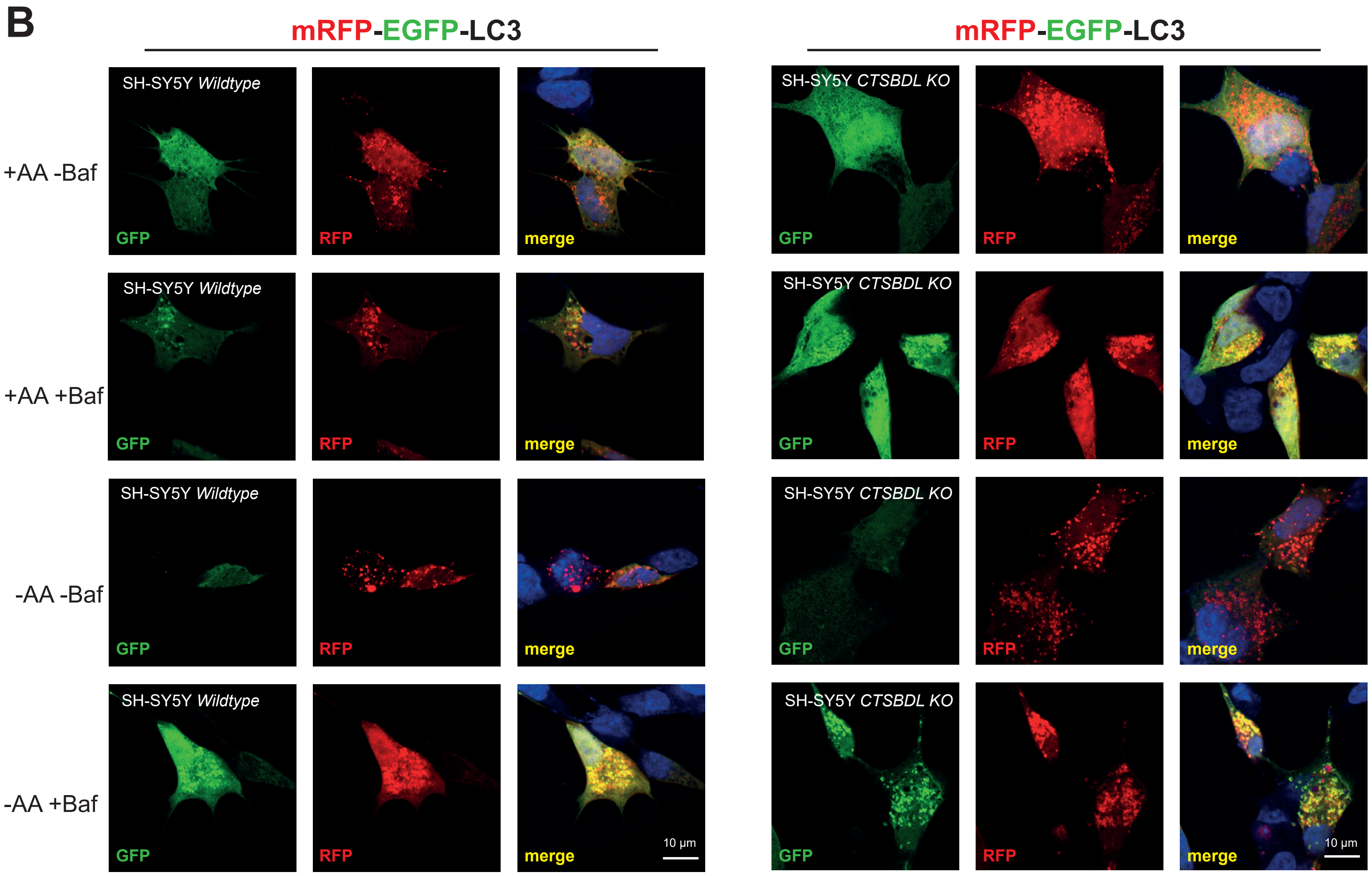

Supplementary Figure 3. Fusion of autophagosomes with lysosomes is unaffected in the Hela CTSBDLZ and SH-SY5Y CTSBDL-deficient cells. Respective immunofluorescence images of tandem mRFP-EGFP-LC3-transfected Hela WT and Hela CTSBDLZ- deficient cells (A) and SH-SY5Y WT and CTSBDL-deficient cells (B). An autophagy assay was conducted for 3 h. Cells were incubated using basal EBSS and essential and non-essential amino acids mixture (AA; first row), with additional lysosomal inhibitor Bafilomycin A1 (BafA1, 500 nM; second row), with EBSS only (starved, third row) and starvation conditions with BafA1 (fourth row). Compared to wildtype cells, HeLa CTSBDLZ and SH-SY5Y CTSBDL-deficient cells show a high autophagy rate under basal conditions. At the same time, additional incubation with BafA1 leads to a complete blockade of autophagy in both wildtype and multi-CTS-deficient cells. All signals were captured at 594 nm for RFP and 488 nm for EGFP. Scale bar: 10 μm

Supplement Figure 4

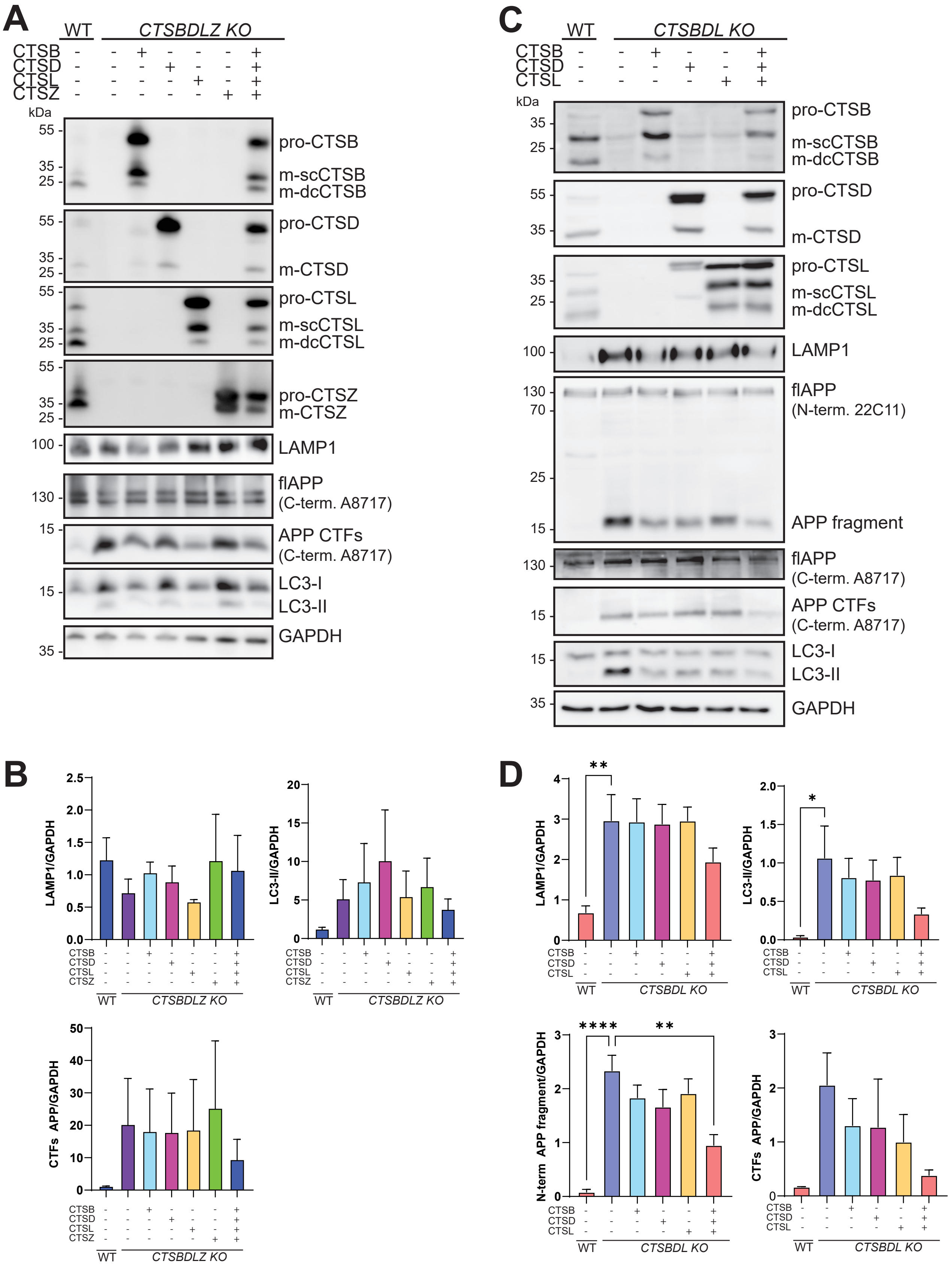

Supplementary Figure 4. CTS-re-transfection only partially rescues lysosomal biology and function. Immunoblot analysis and respective quantification of LAMP1, APP (N-/C-terminal fragments), and LC3 in HeLa WT, CTSBDLZ-deficient, CTSBDLZ-deficient cells, retransfected with either CTS B, D, L, Z or all CTSs jointly (A and B) and SH-SY5Y WT, CTSBDL-deficient, and CTSBDL-deficient cells, retransfected with either CTS B, D, L or all CTSs together (C and D). Proteins of interest were normalized against GAPDH. Data represent the mean ± SEM. **** p<0.0001.
